# Multifactorial bioengineering in the tendon context to maintain tenocyte phenotype and to direct dermal fibroblasts towards tenogenic lineage

**DOI:** 10.1016/j.bioactmat.2025.11.008

**Published:** 2025-12-03

**Authors:** Adrian Djalali-Cuevas, Mandy Rettel, Frank Stein, Mikhail Savitski, Ioannis Gkiatas, Anastasios Korompilias, Ioannis Skoufos, Athina Tzora, Nikitas Prassinos, Nikolaos Diakakis, Dimitrios I. Zeugolis

**Affiliations:** a Laboratory of Animal Science, Nutrition and Biotechnology, School of Agriculture, University of Ioannina, Arta, Greece; b School of Veterinary Medicine, Aristotle University of Thessaloniki, Thessaloniki, Greece; c Proteomics Core Facility, European Molecular Biology Laboratory (EMBL), Heidelberg, Germany; d Department of Orthopaedic Surgery, School of Medicine, University of Ioannina, Ioannina, Greece; e Regenerative, Modular & Developmental Engineering Laboratory (REMODEL), Charles Institute of Dermatology, Conway Institute of Biomolecular & Biomedical Research and School of Mechanical & Materials Engineering, University College Dublin (UCD), Dublin, Ireland

**Keywords:** Multifactorial bioengineering, Dermal fibroblasts, Tenocytes, Cell microenvironment, Tendon engineering

## Abstract

Considering that cells *in vivo* are simultaneously exposed to multiple signals, multifactorial bioengineering slowly but surely becomes the state of play in controlling cell function *in vitro*. In this context, herein we assessed the simultaneous effect of three bioinspired factors (i.e. anisotropic architecture, macromolecular crowding and growth factor supplementation) to maintain human tenocyte phenotype and to direct human dermal fibroblasts towards tenogenic lineage. Anisotropic architecture induced bidirectional cell and deposited extracellular matrix deposition in both cell types. Immunofluorescence analysis revealed that when growth factor and macromolecular crowding were combined significantly increased collagen types I, III, IV and V deposition in both cell types. Proteomics analysis made apparent that the combination of anisotropic architecture, macromolecular crowding and growth factor supplementation brought about the highest degree of tenogenic phenotype maintenance (in the case of human tenocytes) and tenogenic induction (in the case of human dermal fibroblasts). This work lays the foundations for utilising multifactorial bioengineering in the development of tendon-like assemblies.

## Highlights

• Anisotropic architecture induced bidirectional cell and deposited extracellular matrix deposition.
• Growth factor supplementation and macromolecular crowding increased extracellular matrix deposition.
• Anisotropic architecture, growth factor supplementation and macromolecular crowding brought about the highest degree of tenogenic phenotype maintenance in human tendon cell cultures.
• Anisotropic architecture, growth factor supplementation and macromolecular crowding induced the highest degree of tenogenic induction in human dermal fibroblast cultures.

## Graphical abstract

Assessing anisotropic topography, growth factor supplementation and macromolecular crowding in human dermal fibroblast and human tenocyte cultures.

**Figure:**
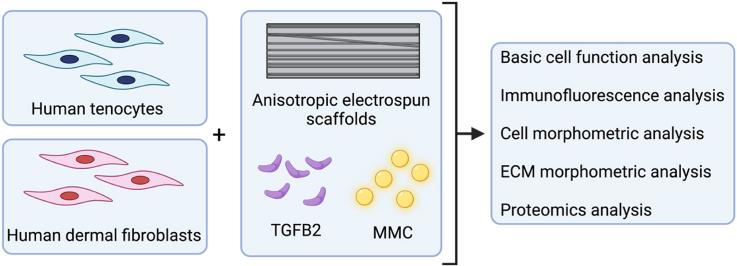

## 1 Introduction

Musculoskeletal conditions are amongst the principal causes of human disability [1], exerting detrimental effects on patients mobility and life quality [2]. In global scale, 6–7 million tendon ruptures occur with estimated healthcare costs in the region of €25–28 billion [3]. Current clinical approaches for critically injured tendons mainly rely on tissue grafts or biomaterials, which, despite significant advances, have yet to completely restore function [4]. Although advanced therapy medicinal products hold promise in tendon engineering [5], very few studies have reached clinical assessment [6], clearly emphasising the need to drive further research efforts into the tendon domain.

Adequate cell numbers and/or appropriate differentiation methodologies are of uttermost importance for successfully development of clinically- and commercially-relevant cell-based therapies. Given their multilineage differentiation potential, mesenchymal stromal cells are an attractive cell source for regenerative strategies [7]. However, their effective tenogenic differentiation is still elusive [8] and their potential for ectopic bone formation [9] hamper subsequent application in tendon engineering. Pluripotent stem cells also hold the potential to differentiate towards the tenogenic lineage [[10], [11], [12]]. However, the associated tumorigenicity and ethical issues impose significant constraints for their clinical translation [13]. Responsible for the production of a highly specialised extracellular matrix (ECM), human tenocytes (hTCs) are the main cell population of tendons and thus, inherent to tendon engineering. However, donor-site morbidity [14], their scarcity within tendon tissue [15] and dedifferentiation issues during *in vitro* culture [16,17] reduce their potential for therapeutic development. Leveraging their ECM-building capabilities, human dermal fibroblasts (hDFs) have also been investigated for tendon regeneration [18]. Despite the positive outcomes attained, similarly to TCs, DFs produce tissue constructs exhibiting inferior mechanical properties than native tendons [[19], [20], [21], [22], [23], [24], [25], [26]].

Originally achieved by cellular transfection of lineage-specific transcription factors [27], direct cell reprogramming enables converting terminally differentiated cells into desired cell populations, bypassing a pluripotent state [28]. In quest of increased safety and efficiency, alternatives to genetic manipulation (i.e. small molecules, growth factors, microRNAs and extracellular vesicles) are actively being explored for direct cell reprogramming [[29], [30], [31], [32]]. Given their prominent role in cell fate determination [[33], [34], [35], [36], [37], [38]], tissue-specific microenvironments are an emerging trend in regenerative medicine [[39], [40], [41]], showing promise in cellular differentiation and reprogramming [[42], [43], [44], [45], [46], [47], [48], [49]]. Tendon resident cells are subjected to a complex physicochemical microenvironment *in vivo*, which is frequently imitated *in vitro* to promote tenogenic induction to cultured cells [[50], [51], [52]]. Based on promising [[53], [54], [55], [56], [57]] but frequently insufficient (e.g. surface topography on too rigid substrates induced hTC trans-differentiation [58]; high density cultures only transiently induced tenogenic induction of hDFs [59]; mechanical overload induced autophagy and hTC apoptosis [60]) outcomes of single-factor approaches, multifactorial approaches, of variable complexity, are progressively taking off for improved tenogenic phenotype either maintenance or induction and tendon healing [[61], [62], [63], [64], [65], [66]].

With these in mind, we selected anisotropic architecture, growth factor supplementation and macromolecular crowding (MMC) as means to develop an effective, yet scalable multifactorial approach to maintain hTC phenotype and to direct hDFs towards tenogenic lineage. Anisotropic architecture, induced through electrospinning, was selected considering the native fibrous tendon architecture and the ability of such scaffolds to preserve/induce tendon phenotype *in vitro* [[67], [68], [69]] and promote functional neo-tendon formation *in vivo* [[70], [71], [72]]. As physiochemical cues are not potent regulators of hDF trans-differentiation [73], growth factors are in general preferred to induce a specific lineage (e.g. insulin growth factor 1 has been used to induce chondrogenic differentiation of an adult female rabbit DF cell line [74]; transforming growth factor beta 1, TGFB1, has been shown to induce osteogenic differentiation of hDFs [75]). Here, we selected TGFB2 as it is involved in the commitment of mesodermal cells [76] and mesenchymal stromal cells [77] towards tendon lineage. MMC was selected as it is the most efficient way to enhance and accelerate ECM deposition [[78], [79], [80]], with well-established beneficial effects in TC cultures (alone or in combination with other *in vitro* microenvironment regulators) [[81], [82], [83], [84], [85]].

## 2 Materials and methods

### 2.1 Materials and reagents

Unless stated otherwise, chemical compounds and cell culture media and supplements were purchased from Sigma-Aldrich (Greece). Cell culture supplies were procured from Sarstedt (Greece). Mimetix® aligned (97.7 ± 1.3 %) electrospun FDA-approved medical grade poly-L-lactide (PLLA) fibres (fibre diameter: 2 μm, scaffold thickness: 2–4 μm, scaffold density: 130 fibres/mm) were purchased from The Electrospinning Company (United Kingdom). Fig. S1 provides brightfield microscopy images of the Mimetix® scaffolds before and after preconditioning with 20 % ethanol, washing with phosphate buffered saline (PBS) and incubation with basal medium, as per manufacturer's instructions. The human recombinant TGFB2 (rhTGFB2) was purchased from R&D Systems (United States of America). The λ-carrageenan (Viscarin® GP 109 NF) was used as MMC agent, as it has been shown to induce the highest ECM deposition in the shortest period of time without affecting basic cellular function in a wide range of cell populations [[86], [87], [88], [89], [90], [91]] and it was provided by FMC Corporation (United States of America).

### 2.2 Cell culture

Cryopreserved hDFs (PCS-201-012) were purchased from the American Type Culture Collection (ATCC, United Kingdom). Patient-donated healthy hamstring tendons were sourced from the University Hospital of Ioannina (Greece), following ethical approval and informed consents obtention. hTCs were isolated from hamstring tendons following the explant culture approach [84]. Three different donors per cell type (details are provided in Table S1) were used for this study. Cell cultures were performed at 37 °C and 5 % CO_2_ humidified atmosphere. Dulbecco's modified Eagle medium (4.5 g/l glucose) supplemented with 10 % foetal bovine serum, 100 μg/ml streptomycin and 100 units/ml penicillin (basal medium), was employed for culturing hDFs and hTCs. At passage 4, hTCs and hDFs were seeded at 25,000 cells/cm^2^ in tissue culture plates containing Mimetix® aligned PLLA fibres and cultured for 24 h in basal medium to ensure adequate cell adhesion. Subsequently, different experimental conditions were introduced by culturing cells with basal medium supplemented with 100 μM L-ascorbic acid 2-phosphate (termed PLLA group) or 100 μM L-ascorbic acid 2-phosphate + 2 ng/ml rhTGFB2 (termed + TGFB2 group) or 100 μM L-ascorbic acid 2-phosphate + 10 μg/ml of λ-carrageenan (termed + MMC group) or 100 μM L-ascorbic acid 2-phosphate + 10 μg/ml of λ-carrageenan + 2 ng/ml rhTGFB2 (termed + MMC + TGFB2 group). The 100 μM L-ascorbic acid 2-phosphate and the 10 μg/ml λ-carrageenan concentrations were selected based on a previous publication [90]. With respect to the rhTGFB2 concentration, over the years growth factors have been used at concentrations as low as 0.1 ng/ml [92] to as high as 1000 ng/ml [93]. As TGF isoforms have been shown to be effective at as low concentrations as 2 ng/ml (B1 [94], B2 [95], B3 [96]), this concentration was used herein. Media were replaced with fresh respective media every 3 days. Analyses were performed 4, 7 and 10 days after cell seeding.

### 2.3 Cell proliferation, viability and metabolic activity analyses

Nuclear fluorescent imaging was employed to analyse cell proliferation. Briefly, samples were washed thrice with Hank's Balanced Salt Solution (HBSS), fixed for 15 min with ice-chilled 2 % paraformaldehyde (PFA) in PBS and washed thrice with PBS. Nuclei were subsequently stained for 5 min with 0.5 μg/ml 4′,6-diamidino-2-phenylindole (DAPI) in methanol. After three PBS washes, samples were visualised with an Olympus IX73 inverted fluorescence microscope (Olympus Corporation, Japan). Cell numbers were quantified using ImageJ software (National Institute of Health, United States of America). Briefly, five images were taken per sample, converted to 16-bit and processed with StarDist 2D plugin using standard parameters, as has been described previously [97]. To estimate nuclei numbers per image, individual nuclei were segmented as separate region of interest and the number of regions of interest were counted. Cell numbers per sample were calculated as the average number of nuclei per corresponding five images. Cell numbers in all experimental conditions and time points were normalised to hTCs PLLA day 4 group.

Live and dead cells were assessed by fluorescent staining with 4 μM calcein-AM and 2 μM ethidium homodimer in HBSS [26]. In brief, after three washes with HBSS, staining solution was applied to the samples for 30 min at 37 °C under 5 % CO_2_ humidified atmosphere. Samples were subsequently washed with HBBS and visualised with an Olympus IX73 inverted fluorescence microscope (Olympus Corporation, Japan).

Spectrophotometric analysis of resazurin reduction was employed to assess cell metabolic activity [26]. Samples were washed with HBSS before the addition of a 44 μM solution of resazurin in HBSS. Incubation was performed for 3 h at 37 °C under 5 % CO_2_ humidified atmosphere. Resazurin solution and HBSS incubated in empty wells were employed as spectrophotometric references. Following incubation, supernatants (100 μl) from each sample were measured on a clear plate at 550 nm and 595 nm using a Biotek® Synergy™ HT microplate reader (Biotek Instruments, United States of America). Absorbance values were employed for calculating the % of resazurin reduction.

### 2.4 Immunofluorescence analysis

Immunofluorescence analysis was conducted to assess deposited ECM as per established protocol [26]. Table S2 provides the details (i.e. antigen, species, clonality, dilution, product number, manufacturer) of the primary and secondary antibodies used. After three HBSS washes, samples were fixed for 15 min with ice-chilled 2 % PFA in PBS and washed thrice again with PBS. Blocking was carried out for 30 min using 3 % bovine serum albumin (BSA) in PBS. Incubation with primary antibodies diluted in PBS was subsequently performed for 1.5 h and followed by three PBS washes. Secondary antibodies diluted in PBS were subsequently applied to the samples for 30 min. Collagen type I (COL I) was detected using Alexa Fluor® Plus 555 as conjugated fluorochrome, whilst collagen types III, IV, V and VI (COL III, IV, V and VI) were detected with Alexa Fluor® Plus 488. After three PBS washes, samples were post-fixed with 2 % PFA in PBS for 15 min and washed again with PBS. Nuclei were stained with 0.5 μg/ml DAPI in methanol for 5 min. After three PBS washes, samples were visualised with an Olympus IX73 inverted fluorescence microscope (Olympus Corporation, Japan). Fluorescence intensity analyses were performed using ImageJ software (National Institute of Health, United States of America). Microscope and camera settings remained constant during all imaging to ensure measurement consistency. To determine sample autofluorescence, controls with no primary antibody were imaged (blanks). Five images were taken per sample, converted to 8-bit and mean grey values were obtained per image. Fluorescence intensities per sample were calculated as the average value per corresponding five images. Mean values per experimental conditions and time points were blank-subtracted and expressed as absolute numbers.

Detection of tenomodulin (TNMD) and alpha-smooth muscle actin (aSMA) was performed via immunofluorescent staining, as has been previously described [26]. Briefly, after three HBSS washes, samples were fixed for 15 min with ice-chilled 2 % PFA in PBS. After three PBS washes, permeabilisation was performed with 0.1 % Triton X-100 in PBS for 10 min. Blocking was performed with 5 % BSA and 0.02 % Triton X-100 in PBS for 1.5 h. After blocking, primary antibodies were applied to the samples for 2.5 h, diluted in 3 % BSA and 0.02 % Triton X-100 in PBS. Staining with F-actin was performed where appropriate by adding 50 nM rhodamine-phalloidin (Invitrogen, Ireland) to the primary antibody solution. After three washes with 0.5 % BSA and 0.02 % Triton X-100 in PBS (PBT), secondary antibodies were applied to the samples for 30 min, diluted in 3 % BSA and 0.02 % Triton X-100 in PBS. TNMD was detected using Alexa Fluor® Plus 488 as conjugated fluorochrome, whilst aSMA was detected using Alexa Fluor® Plus 555. After three washes with PBT, samples were post-fixed for 15 min with 2 % PFA in PBS and washed once again with PBT. A solution of DAPI (0.5 μg/ml) in PBT was employed to stain nuclei for 5 min. Sample visualization was performed, after three PBS washes, with an Olympus IX73 inverted fluorescence microscope (Olympus Corporation, Japan). ImageJ software (National Institute of Health, United States of America) was employed for image analysis. To ensure consistent measurements, microscope and camera settings remained constant during all imaging. To determine sample autofluorescence, controls with no primary antibody were imaged (blanks). Five images were taken per sample. Cell numbers per image were determined as described in the cell proliferation section. For TNMD analysis, images were converted to 8-bit and mean grey values were obtained per image and blank-subtracted. Fluorescence intensity per image was normalised to cell numbers. Normalised fluorescence intensity per sample was calculated as the average value per corresponding five images. TNMD fluorescence intensity per cell was normalised to hTCs PLLA day 4 group in all experimental conditions and time points. For aSMA analysis, the number of myofibroblasts was counted per image, and values per sample were calculated as the average numbers per corresponding five images. Myofibroblast numbers were expressed as a percentage of the total cells determined per sample.

### 2.5 Cell and ECM morphometric analyses

Cytoskeletal and nuclear fluorescent co-staining was performed as per established protocol [26]. Briefly, samples were washed thrice with HBSS, fixed for 15 min with ice-chilled 2 % PFA in PBS, and washed thrice again with PBS. Permeabilisation was performed with 0.1 % Triton X-100 in PBS for 10 min. Blocking was performed with 5 % BSA and 0.02 % Triton X-100 in PBS during 1.5 h. To stain actin filaments (F-actin), rhodamine-phalloidin (50 nM) (Invitrogen, Ireland) in 3 % BSA and 0.02 % Triton X-100 in PBS was applied to the samples for 2.5 h. After three washes with PBT, post-fixation was performed for 15 min with 2 % PFA in PBS. After an additional wash with PBT, nuclei were stained for 5 min with 0.5 μg/ml DAPI in PBT and washed thrice with PBS. Immunofluorescent staining for COL I, III, IV, V and VI was performed as described in the previous section. Sample visualization was performed with an Olympus IX73 inverted fluorescence microscope (Olympus Corporation, Japan). To obtain quantitative measurements of nuclear, cytoskeletal and collagen orientation, the corresponding fluorescent images were processed using ImageJ software (National Institute of Health, United States of America) equipped with the OrientationJ plugin [98,99].

### 2.6 Proteomics analysis

Proteomics analysis was performed as has been previously described [26], with minor modifications. Hamstring tendon tissue (termed TT group), as well as hTCs and hDFs cultured with basal medium supplemented with 100 μM L-ascorbic acid 2-phosphate in standard tissue culture polystyrene plates (termed hTCs TCP and hDFs TCP groups), were included in the analysis as positive and negative controls, respectively. Briefly, tendon samples were finely sliced under liquid N_2_ and cell layers were gently detached from the culture surfaces after 10 days of culture. Samples were lysed with 4 % SDS in 50 mM Tris-HCl pH 7.6. Protein concentration was determined with the Pierce™ BCA Protein Assay Kit (ThermoFisher, United States of America), using BSA as standard. The SP3 protocol [100] was employed for sample processing, followed by overnight on-bead digestion at 37 °C with sequencing-grade trypsin (Promega, United States of America). TMT-11plex™ Isobaric Label Reagent (ThermoFisher, United States of America) was employed for peptide labelling. An OASIS® HLB μElution Plate (Waters Corporation, United States of America) was used for sample clean up. An Agilent 1200 Infinity HPLC system (Agilent Technologies, United States of America), equipped with a Gemini® C18 column (Phenomenex, United States of America), was employed for offline high-pH reverse phase fractionation, resulting in 12 fractions [101].

Quantitative proteomic analysis was performed by nano-liquid chromatography coupled to tandem mass spectrometry (nLC-MS/MS). An UltiMate™ 3000 RSLC nano system (Dionex Corporation, United States of America) incorporating an Acclaim™ PepMap™ 100C18 trapping cartridge (ThermoFisher, United States of America) and a nanoEase™ M/Z HSS T3 C18 analytical column (Waters Corporation, United States of America) was employed for in-line peptide fractionation. An Orbitrap™ Q Exactive™ Plus hybrid quadrupole mass spectrometer (ThermoFisher, United States of America) was directly coupled to the outlet of the analytical column through a Nanospray Flex™ Ion Source (ThermoFisher, United States of America). A Pico-Tip® Emitter with an applied spray voltage of 2.3 kV was employed to introduce the peptides into the mass spectrometer. Data-dependent acquisition was employed for mass spectra collection. Data processing was performed with IsobarQuant [102] and Mascot (v2.2.07) [103]. Protein identification was performed against a Uniprot *Homo sapiens* proteome database (UP000005640) including reversed sequences and common contaminants [104]. The mass error tolerance was set to 10 ppm, and 0.02 Da, for the for the full scan (MS1), and MS/MS (MS2) spectra, respectively. Trypsin was selected as protease with a maximum allowance of two missed cleavages. For protein identification, at least two unique peptides were required with a minimum peptide length of seven amino acids. False discovery rate (FDR) was set to 0.01 on peptide and protein level.

### 2.7 Bioinformatics analysis

R programming language [105] was used for nLC-MS/MS data processing. Only proteins identified in at least two replicates per group were kept for downstream analysis. The limma package [106] was employed for batch-effect removal. Normalisation and missing values imputation were performed using the vsn [107] and Msnbase [108] packages, respectively. The limma package [106] was employed for testing differential protein abundance. Significant differences between protein levels (‘hits’) were determined when a 2-fold change (FC) was detected with an FDR below 0.05. Proteins hits in tendon tissue were analysed with Genevestigator (v9.14.0) (NEBION AG, Switzerland) [109]. These were considered contaminants when their corresponding mRNA expression values were low in tendon tissue (i.e. < 10 %), and found to be expressed at the mRNA level (i.e. > 60 %) in muscle and fat; or at the protein level in blood [110,111]. Principal component analysis was performed with BioVinci (v2.0, BioTuring, United States of America). Venn diagrams were produced with Dr. Van de Peer lab's Bioinformatics & Evolutionary Genomics web resource [112]. Gene ontology (GO) annotations were performed using PANTHER (v18.0) statistical over-representation test [113,114], and plotted with SRplot [115]. Gene Set Enrichment Analysis (v4.3.2) [116] was performed using the protein hits in tendon tissue versus hTCs TCP and hDFs TCP, as gene sets for hTCs and hDFs analyses, respectively. Matrisome AnalyzeR [117] was employed for the identification of ECM proteins within the proteomics datasets. Heatmaps were created with Heatmapper [118].

### 2.8 Statistical analysis

The proteomics work was performed in three biological replicates. All other experiments were performed in three technical replicates. Mean values ± standard deviations were used to represent numerical data. Statistical analysis was performed with SigmaPlot (v12) (Systat Software, Inc, United States of America), except otherwise stated. Statistically significant differences between group mean values were detected by One-way ANOVA followed by Tukey's HSD post-hoc test. The Shapiro-Wilk and Levene's tests were used for normality and equal variance testing, respectively. When parametric analysis assumptions were violated, the Kruskal-Wallis test was performed followed by the Student-Newman-Keuls post-hoc test. Except otherwise stated, differences between groups were considered statistically significant when p-values were below 0.05.

## 3 Results

### 3.1 Cell proliferation, viability and metabolic activity analyses

Cell proliferation analysis (Fig. S2) revealed that TGFB2 significantly (p < 0.05) increased proliferation of hDFs and hTCs (alone and in combination with MMC), after 7 and 10 days of culture and MMC did not significantly (p > 0.05) affect cell proliferation of hTCs or hDFs. Proliferation in hDFs was significantly (p < 0.05) higher than in hTCs (apart from PLLA and +TGFB2 groups at day 4, p > 0.05) at all time points and conditions. As a function of time in culture, cell proliferation significantly (p < 0.05) increased under all experimental conditions in hDFs and in +TGFB2 and +MMC + TGFB2 conditions in hTCs. Cell viability analysis (Fig. S3) did not reveal any qualitative differences between the groups and all conditions afforded physiological cell growth. Assessment of cell metabolic activity (Fig. S4) revealed no significant (p > 0.05) differences (apart from day 4, +MMC + TGFB2 groups significantly, p < 0.05, lower than respective PLLA groups; and day 10 hTCs + MMC + TGFB2 significantly, p < 0.05, lower than respective PLLA group) between experimental conditions. In hDFs + MMC + TGFB2, metabolic activity significantly (p < 0.05) increased from day 4 to day 7 and day 10.

### 3.2 Immunofluorescence analysis

**Fig. 1:**
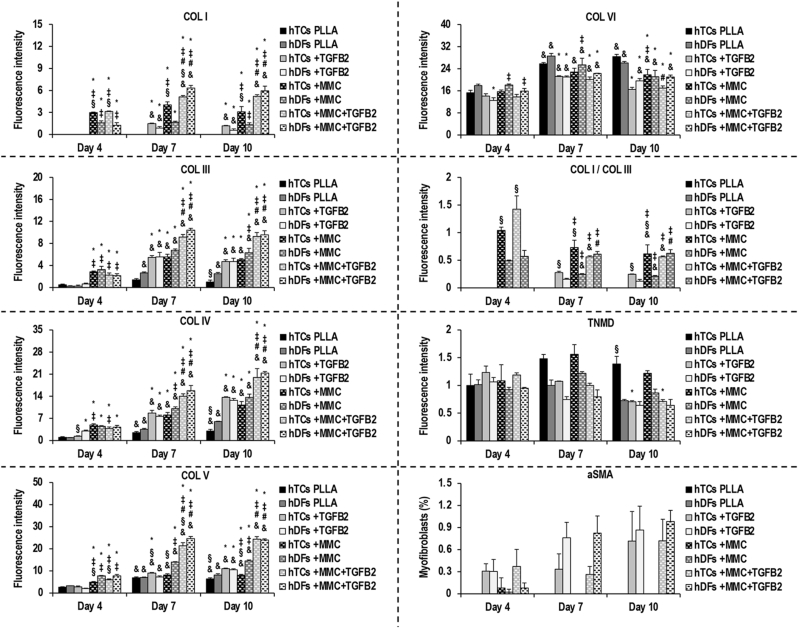
Immunofluorescence intensity analysis for COL I, III, IV, V, VI, COL I/COL III ratio, TNMD and aSMA in hTCs and hDFs after 4, 7 and 10 days of culture under PLLA, +TGFB2, +MMC and +MMC + TGFB2 conditions. (∗p < 0.05 vs respective PLLA group at the same time point; ‡p < 0.05 vs respective + TGFB2 group at the same time point; #p < 0.05 vs. respective + MMC group at the same time point; §p < 0.05 between cell types at the same time point and condition; &p < 0.05 vs. respective condition at day 4). N = 3.

Immunofluorescence (Figs. S5–S11) and image intensity (Fig. 1, Tables S3–S5) analyses revealed that in the PLLA groups, the deposition of COL I, COL III, COL IV and COL V ranged from non-detectable to low in both hTCs and hDFs at all time points. In general, at day 7 and day 10, supplementation of hTC and hDF cultures with TGFB2 significantly (p < 0.05) increased the levels of COL I, COL III, COL IV and COL V (except in hDFs at day 7, p > 0.05); and of COL IV at day 4 in hDFs. At all time points, MMC significantly (p < 0.05) increased the deposition of COL I, COL III, COL IV and COL V (except in hTCs at day 7, p > 0.05) in both hTC and hDF cultures. The deposition of COL I, COL III, COL IV and COL V in hTCs and hDFs was significantly (p < 0.05) increased by the combination of TGFB2 and MMC, with respect to the corresponding PLLA groups, at all time points; and was significantly (p < 0.05) higher than the corresponding + TGFB2 and +MMC groups at day 7 and day 10. TGFB2 supplementation significantly (p < 0.05) reduced the deposition of COL VI in hTCs and hDFs, alone or in combination with MMC, at day 7 and day 10; as well as alone in hDFs at day 4. MMC did not significantly (p > 0.05) increase COL VI deposition at any time point and condition, but significantly (p < 0.05) decreased COL VI deposition in hTCs and hDFs at day 10. Between hTCs and hDFs, COL I deposition was significantly (p < 0.05) higher in hTCs at days 4, 7 and 10 in +MMC groups, and at day 4 and in the +MMC + TGFB2 group. At day 7, COL I deposition in +MMC + TGFB2 groups was significantly (p < 0.05) higher in hDFs than in hTCs, but no significant (p > 0.05) differences were longer detected at day 10. In the case of COL III, differences were only detected between PLLA groups at day 10, being levels in hDFs significantly (p < 0.05) higher than in hTCs. For COL IV, hDFs deposited significantly (p < 0.05) higher levels than hTCs at day 4 and day 10, in +TGFB2 and PLLA conditions, respectively. Deposition of COL V was significantly (p < 0.05) higher in hTCs than in hDFs only under + TGFB2 conditions at day 7. Conversely, hDFs deposited significantly (p < 0.05) higher COL V than hTCs at day 10 under PLLA conditions, at days 4, 7 and 10 under + MMC conditions, and at day 4 and day 7 but not at day 10 (p > 0.05) under + MMC + TGFB2 conditions. No significant (p > 0.05) differences were detected between hTCs and hDFs COL VI deposition at any time point or condition. As a function of time in culture, significant (p < 0.05) increases were detected from day 4 to day 7 and day 10, in hTCs and hDFs under all experimental conditions, for the deposition of COL I (apart from PLLA and +MMC groups, p > 0.05), COL III (except hTCs PLLA, p > 0.05), COL IV, COL V and COL VI (except hTCs + TGFB2 and hTCs + MMC + TGFB2 at day 10, p > 0.05). The COL I/COL III fluorescence intensity ratio, at days 7 and 10, was significantly (p < 0.05) higher in +MMC and +MMC + TGFB2 groups than in +TGFB2 groups, for both hTCs and hDFs, and significantly (p < 0.05) higher in the +MMC + TGFB2 group than in the +MMC group, only in hDFs. Under + TGFB2 and +MMC conditions, the COL I/COL III ratio was significantly (p < 0.05) higher in hTCs than in hDFs, at all time points. Under + MMC + TGFB2 conditions, the COL I/COL III ratio was significantly (p < 0.05) higher in hTCs than in hDFs at day 4, but significant differences were no longer detected at days 7 and 10 (p > 0.05). As a function of time in culture, the COL I/COL III ratio significantly (p < 0.05) decreased from day 4 to day 7 in hDFs + MMC and hTCs + MMC + TGFB2, and from day 4 to day 10 in both + MMC groups and in hTCs + MMC + TGFB2.

Expression of TNMD was detected in hDFs and hTCs at all time points and conditions. Between experimental conditions, significant differences were only detected in hTCs at day 10, being TNMD expression in the PLLA group, significantly (p < 0.05) higher than in the respective + TGFB2 and +MMC + TGFB2 groups. Between cell types, only under PLLA conditions at day 10, hTCs expressed significantly (p < 0.05) higher TNMD levels than hDFs. As a function of time in culture, no significant (p > 0.05) differences were detected for any experimental condition. By means of aSMA immunofluorescence, myofibroblast trans-differentiation was detected in +TGFB2 and +MMC + TGFB2 groups, at all time points, and in +MMC groups, at day 4. However, the extent of myofibroblast trans-differentiation was marginal (<1 %) in every case, and no significant (p > 0.05) differences were detected between experimental conditions, cell types or time points.

### 3.3 Cell and ECM morphometric analyses

Qualitative (Fig. S12) and quantitative (Figs. S13–S14) hTCs and hDFs morphometric analyses revealed that both nuclei and cytoskeleton were aligned in the direction of the aligned Mimetix® PLLA fibres with no significant (p > 0.05) differences between the experimental groups at any time points. Quantitative morphometric analysis of the deposited COL I, COL III, COL IV, COL V and COL VI in hTC and hDF cultures (Fig. S15) also revealed ECM orientation in the direction of the aligned Mimetix® PLLA fibres with no significant (p > 0.05) differences between the experimental groups at any time points.

### 3.4 Proteomics analysis

**Fig. 2:**
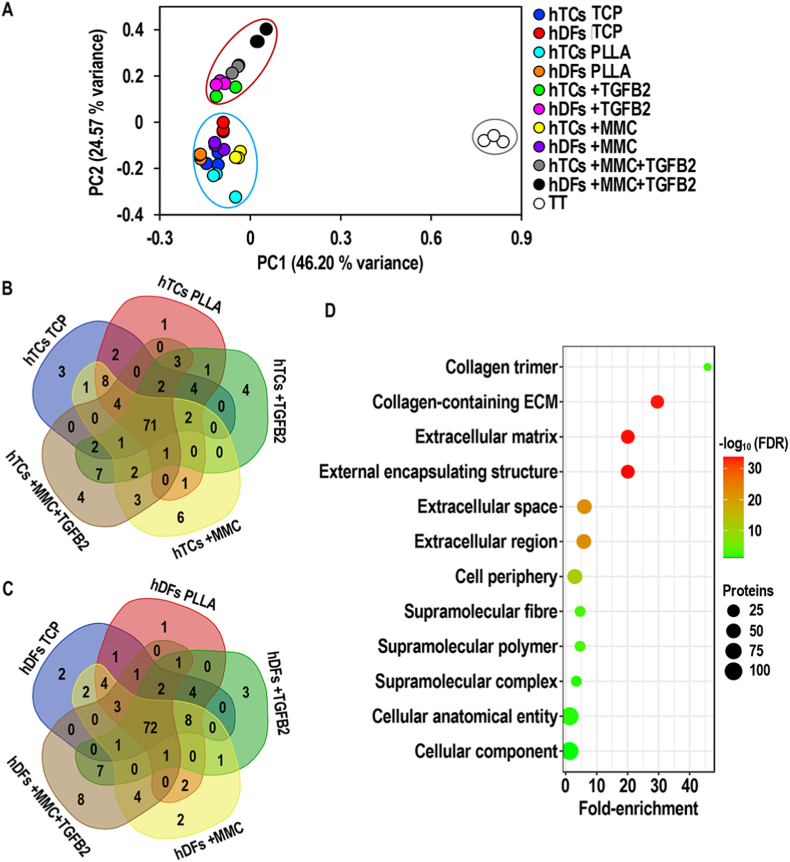
Principal component analysis (**A**) of the quantitative proteomic data obtained from hTCs and hDFs after 10 days of culture under TCP, PLLA, +TGFB2, +MMC and +MMC + TGFB2 conditions, as well as from TT samples. Venn diagram representing the top 100 protein hits in TT group vs. hTCs (**B**) and hDFs (**C**) cultured under PS, PLLA, +TGFB2, +MMC and +MMC + TGFB2 conditions. Gene ontology analysis (**D**) of the top 100 protein hits in TT group vs. hTCs and hDFs cultured under PS, PLLA, +TGFB2, +MMC and +MMC + TGFB2 conditions. N = 3.

**Fig. 3:**
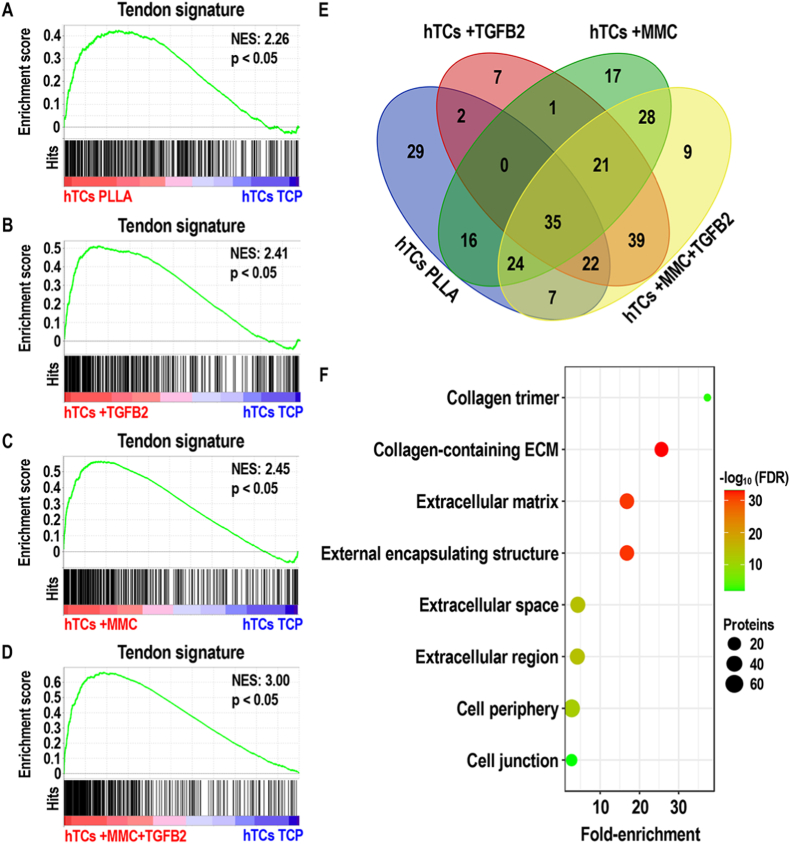
Gene set enrichment analysis of the quantitative proteomic profile of hTCs cultured for 10 days under PLLA (**A**), +TGFB2 (**B**), +MMC (**C**), and +MMC + TGFB2 (**D**) conditions. All contrasts were performed against hTCs cultured for 10 days under TCP conditions. A gene set containing all protein hits in TT group vs hTCs TCP group (tendon signature) was used for all analyses. Venn diagram summarizing the different core enrichment proteins identified in PLLA, +TGFB2, +MMC and +MMC + TGFB2 groups by means of gene set enrichment analysis (**E**). GO analysis of the core enrichment proteins identified in +MMC + TGFB2 group by means of gene set enrichment analysis (**F**). (NES: normalised enrichment score). N = 3.

A total of 5527 proteins were identified in the full proteomic dataset (**File S1**). Principal component analysis (Fig. 2A) of the quantitative proteomics data revealed that samples were grouped in three main clusters. One cluster contained all samples from the TT group, a second cluster contained all samples from the +TGFB2 and +MMC + TGFB2 groups and a third one contained all samples from the TCP, PLLA and +MMC groups. Venn diagrams of the top 100 protein hits in TT group versus hTCs (Fig. 2B) or hDFs (Fig. 2C) cultured under TCP, PLLA, +TGFB2, +MMC and +MMC + TGFB2 conditions (**File S2**), revealed that a great proportion of these (i.e. 71 for hTCs and 72 for hDFs) were common for all comparisons. GO annotation (Fig. 2D) of the top 100 protein hits in TT group versus hTCs and hDFs cultured under TCP, PLLA, +TGFB2, +MMC and +MMC + TGFB2 conditions revealed significant (FDR <0.05) enrichment of extracellular- and cytoskeletal-related proteins within them. Following gene set enrichment analysis, the quantitative proteomic profile of hTCs cultured under PLLA, +TGFB2, +MMC and +MMC + TGFB2 demonstrated a statistically significant (p < 0.05) positive correlation with the corresponding TT signature gene set, when contrasted against hTCs cultured on TCP (Fig. 3A–D). Additionally, the normalised enrichment score (NES) was the lowest (NES: 2.26) in the PLLA group; similar between + TGFB2 (NES: 2.41) and +MMC (NES: 2.45) groups; and the highest (NES: 3.00) in the +MMC + TGFB2 group. The hTC core enrichment proteins of the PLLA, +TGFB2, +MMC and +MMC + TGFB2 are detailed in **Files S3-S6** and summarised in Fig. 3E. GO annotation of the core enrichment proteins from each gene set enrichment analysis, revealed that ECM proteins were significantly (FDR <0.05) enriched within the proteins induced by PLLA, +TGFB2, +MMC and +MMC + TGFB2 (highest amount of ECM proteins) conditions. Also, cytoskeletal proteins and cell junction proteins were significantly (p < 0.05) enriched in +MMC and +MMC + TGFB2 groups, respectively (Tables S6–S8, Fig. 3F).

**Fig. 4:**
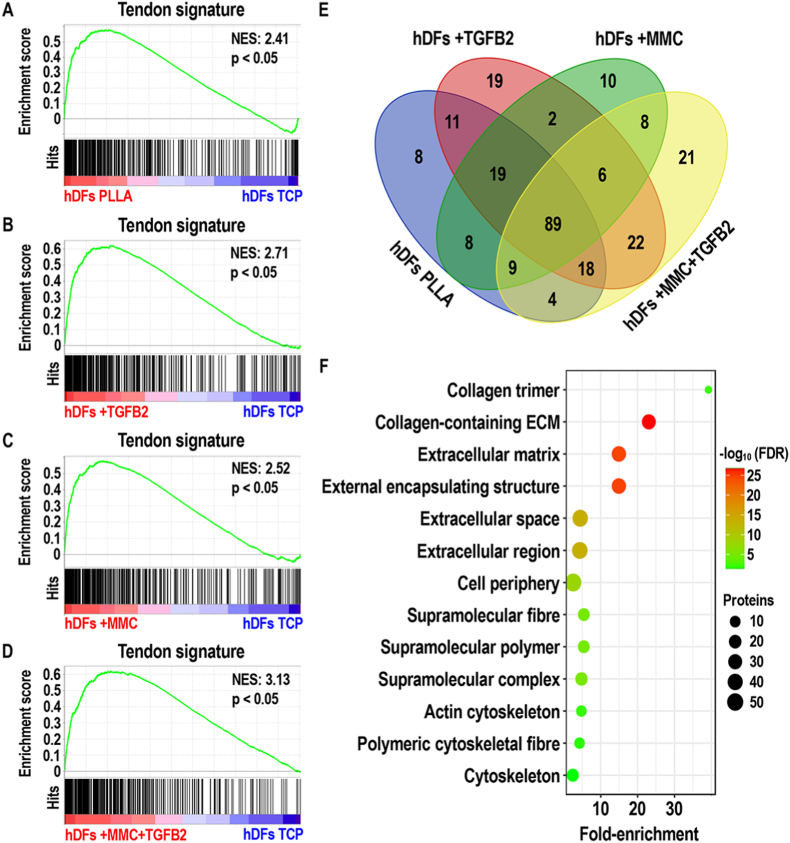
Gene set enrichment analysis of the quantitative proteomic profile of hDFs cultured for 10 days under PLLA (**A**), +TGFB2 (**B**), +MMC (**C**), and +MMC + TGFB2 (**D**) conditions. All contrasts were performed against hDFs cultured for 10 days under TCP conditions. A gene set containing all protein hits in TT group vs hDFs TCP group (tendon signature) was used for all analyses. Venn diagram summarizing the different core enrichment proteins identified in PLLA, +TGFB2, +MMC and +MMC + TGFB2 groups by means of gene set enrichment analysis (**E**). GO analysis of the core enrichment proteins identified in +MMC + TGFB2 group by means of gene set enrichment analysis (**F**). (NES: normalised enrichment score). N = 3.

In the case of hDFs, gene set enrichment analysis of the quantitative proteomic profile of PLLA, +TGFB2, +MMC and +MMC + TGFB2 groups revealed a statistically significant (p < 0.05) positive correlation with the corresponding TT signature gene set, in contrast with hDFs in TCP group (Fig. 4A–D). The NES value was the lowest (NES: 2.41) for the PLLA group; medium for the +MMC (NES: 2.52) and the +TGFB2 (NES: 2.71) groups; and the highest (NES: 3.13) for the +MMC + TGFB2 group. The core enrichment proteins of hDFs cultured under PLLA, +TGFB2, +MMC and +MMC + TGFB2 conditions are provided in **Files S7-S10** and summarised in Fig. 4E. GO annotation of the core enrichment proteins from each gene set enrichment analysis demonstrated that ECM proteins were significantly (FDR <0.05) enriched within the proteins induced by PLLA, +TGFB2, +MMC and +MMC + TGFB2 (highest amount of ECM proteins) conditions. In addition, cytoskeletal proteins and cell junction proteins were significantly (FDR <0.05) enriched within the core enrichment proteins identified in PLLA, +TGFB2, +MMC and +MMC + TGFB2 groups and the +TGFB2 group, respectively (Tables S9–S11, Fig. 4F).

**Fig. 5:**
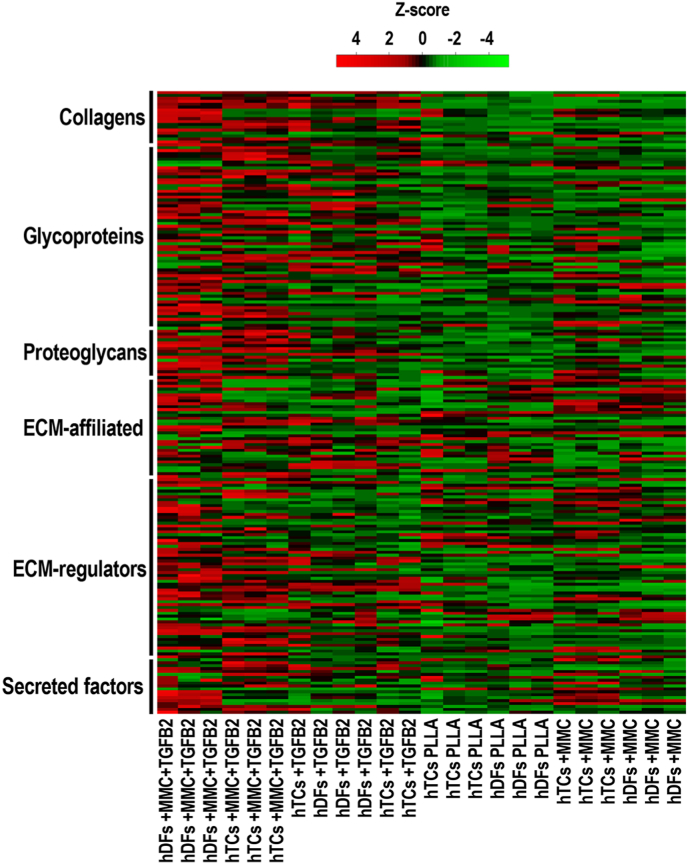
Heatmap displaying the quantitative matrisome of hTCs and hDFs cultured under PLLA, +TGFB2, +MMC and +MMC + TGFB2 conditions for 10 days. Hierarchical clustering was applied to all samples. The heatmap displays the z-scores for every matrisome protein identified, being these grouped into the different matrisome categories. Both hTCs and hDFs clustered first in relation to TGFB2 treatment (alone or in combination with MMC), and ultimately in relation with the experimental conditions to which these were subjected. The + MMC + TGFB2 experimental condition exerted the highest changes into the quantitative matrisome of hTCs and hDFs, increasing ECM protein levels throughout all the categories of the matrisome. N = 3.

Matrisome AnalyzeR analysis (**File S11**) revealed a total of 214 ECM proteins within the full proteomic dataset, with 99 and 115 of which were annotated as core matrisome (classified as 19 collagens, 63 ECM glycoproteins, 17 proteoglycans) and matrisome-associated proteins (classified as 33 ECM affiliated, 62 ECM regulators, 20 secreted factors), respectively. Quantitative matrisome analysis of PLLA, +TGFB2, +MMC and +MMC + TGFB2 groups using Heatmapper (Fig. 5), demonstrated that both hTCs and hDFs samples clustered firstly in relation to TGFB2 treatment (alone or in combination with MMC) and then in relation to the other experimental culture conditions. Regarding the effects of the different experimental treatments on the matrisome of hTCs and hDFs, it was evidenced that + MMC + TGFB2 culture conditions most affected the matrisome of both cell types (i.e. increased the levels of ECM proteins throughout all the categories of the matrisome). Under + TGFB2 culture conditions, a similar effect was noticeable but at a lesser extent than in +MMC + TGFB2 groups. +MMC and PLLA conditions increased the levels of discrete ECM proteins at different matrisome categories.

## 4 Discussion

Complete restoration of tendon function after injury or disease remains an unmet clinical need. Cell-based strategies hold promise for tissue and organ regeneration. However, cell sourcing and tenogenic (de)differentiation, are still a major hurdle for developing advanced therapies for tendons. Direct cell reprogramming can overcome these limitations by converting readily available somatic cells into the cell population of choice. Addressing phenotypic drift can also pose benefits for therapies development. Given that tissue-specific microenvironments have demonstrated to strongly influence cellular phenotype, herein, a tendon microenvironment was engineered *in vitro* and its potential herein to maintain hTC phenotype and/or to direct hDFs towards tenogenic lineage was assessed.

### 4.1 Cell proliferation, viability and metabolic activity analyses

Analysis of cell proliferation, viability and metabolic activity demonstrated that the combination of microenvironmental cues employed for reconstructing the tendon microenvironment *in vitro* (namely anisotropic PLLA microfibres, TGFB2 and MMC) was not detrimental for both hTCs and hDFs. Nonetheless, a slight reduction in cell metabolic activity was observed in MMC-treated conditions, being statistically significant in some + MMC + TGFB2 groups at discrete time points. Previous work has reported similar results upon MMC treatment [26,88] and although the grounds for this observation remain unclear, significant body of evidence discards any possible cytotoxicity exerted by MMC to cultured cells [26,[84], [85], [86], [87], [88]]. In line with previous work [26,119], proliferation was higher in hDFs than in hTCs under all experimental conditions. However, these differences were notably reduced by TGFB2 treatment, which increased proliferation in both cell types.

### 4.2 Immunofluorescence analysis

To put our data into perspective, it is important to remind the reader that all molecules assessed have similar function in both tendon and skin tissues. Indeed, in tendon COL I provides mechanical strength and is responsible for force transmission [120], whilst in skin COL I is responsible for mechanical integrity and elasticity [121]; COL III plays an important role in healing process [122,123]; the ratio of COL I to COL III decreases with aging and pathologies [124,125]; COL IV is basement membrane collagen, in tendon prevents cell extravagation and spontaneous fibrous adhesions [126], whilst in skin structural alterations are associated with several pathologies [127]; COL V regulates physiological assembly, structure and function [128,129]; COL VI regulates physiological assembly [130,131]; although TNMD is primarily associated with physiological tendon development [132], TNMD is highly expressed in adipose tissue [133] and has also been detected in the eye and skin [134]; and aSMA is present in cells of healing tendons [135] and is related to delayed healing in skin [136]. Analysis of collagen deposition by immunofluorescence revealed that both TGFB2 and MMC increased the amounts of COL I, COL III, COL IV and COL V fibres in hTCs and hDFs, whilst their levels remained low or non-detectable under PLLA conditions. Remarkably, in both cell types, the highest deposition of the aforementioned collagen types was cooperatively induced at day 10 by TGFB2 and MMC, likely through enhancing their intracellular synthesis [137,138] and subsequent extracellular deposition [86,87]. It is also worth noting that despite at some time points and conditions, differences in terms of COL I, COL III, COL IV and COL V deposition existed between hTCs and hDFs, these were no longer detected after 10 days of culture under + MMC + TGFB2 conditions. Contrarily, consistent with its non-proteolytic assembly [139], extracellular COL VI fibres were detected in hTCs and hDFs and increased as a function of time in culture, under all experimental conditions. Also, individual and combined treatment with TGFB2 and MMC did not increase COL VI deposition, being the highest levels detected under PLLA conditions. The ratio of COL I/COL III decreased as a function of time in culture in hTCs + MMC, hTCs + MMC + TGFB2 and hDFs + MMC, concordant with the faster deposition of COL I vs. COL III observed in this study. Remarkably, +TGFB2+MMC treatment reverted this effect in hDFs and abolished the differences between cell types detected under all other experimental conditions; which is encouraging given the role of this ratio in tenogenic phenotype [16] and connective tissue mechanics [140]. TNMD was detected (at low levels) in both cell types under all experimental conditions and its levels remained rather constant/were not increased by TGFB2 and/or MMC treatment in any case. We feel that the presence of TNMD is important, as its loss is associated with inferior healing [141]. We also believe that the not increased levels of TNMD may be due to the fact that it is a late tendon marker [142] and appropriate level of homeostasis was not reached within 10 days of culture (longer time point assessed herein). To further substantiate this, one should consider that similarly to our data, in hTC cultures, very low protein (e.g. after 7 [83] and 10 [26] days in culture) and not detected gene (e.g. after 10 [143] and 14 [84] days in culture) TNMD levels have been documented. Although aSMA was detected mainly in TGFB2-treated conditions, it is unlikely that this is indicative of fibrosis, given its marginal abundance (<1 %) [144]. Collectively, these data indicate a physiological tissue assembly when hTCs and hDFs were cultured on anisotropic PLLA scaffolds under MMC and TGFB2 conditions.

### 4.3 Cell and ECM morphometric analyses

Evaluation of qualitative and quantitative nuclei and actin filaments orientation demonstrated the ability of the anisotropic PLLA microfibres to induce, at all experimental conditions and time points, aligned parallel to the orientation of the microfibres nuclei and cytoskeleton in both hTC and hDF cultures. This effect is of remarkable importance given that biomaterial-based mechanical signals are transduced to the nucleus by the cytoskeleton, increasing chromatin accessibility and thus, easing cellular differentiation [39]. In addition, the PLLA microfibres induced bidirectional collagen deposition (where this was possible), enabling therefore the engineering of physiologically relevant tendon microenvironments *in vitro*, mimicking their native cellular and extracellular configuration [15]. Notably, under + MMC + TGFB2 conditions, a dense parallel array of collagen fibres was produced, outperforming previous attempts in terms of fibres alignment [84], quantity [145] and production time [146]. Given that aligned collagen fibres are the main constituent of tendon tissue [50], the herein presented methodology seems promising for engineering tendon microenvironments *in vitro* and developing advanced therapies for tendons using hTCs and hDFs as cell sources.

### 4.4 Proteomics analysis

In terms of quantitative proteomic profile, all cell cultures showed substantial differences with native tendon tissue, as evidenced by principal component analysis. Venn diagrams demonstrated that, in both hTCs and hDFs, the proteins mainly contributing to this divergence were highly overlapping between the different experimental conditions. GO annotation revealed these as ECM- and cytoskeletal-related, consistent with their roles in mechanical load transmission [50] and its cellular sensing [147]; both fundamental for tendon function and homeostasis [50]. These results are of no surprise considering the relatively high ECM-to-cells ratio found in adult tendons [148], as well as the positive correlation between cytoskeletal protein expression and tendon mechanical stimulation [149]; the latter being absent during *in vitro* culture. Despite the highlighted differences with tendon tissue, and contrary to expectations considering the different origins of hTCs and hDFs [150,151], the assessed microenvironmental regulators profoundly modulated the quantitative proteomic profile of both cell types in a similar fashion. Principal component analysis demonstrated sample clustering according to experimental conditions rather than according to cell types; grouping separately TGFB2-treated and non-TGFB2-treated samples. Gene set enrichment analysis substantiated the capacity of tendon-mimicking microenvironmental cues to induce a marked tenogenic phenotype in both hTCs and hDFs. GO analysis of the induced tendon-related proteins under every experimental condition, revealed these mainly consisted in collagens and diverse ECM components, as well as cytoskeletal proteins; both of which exert fundamental roles in tendon function and homeostasis [50,147]. Interestingly, as judged by NES values and the number of tendon-related proteins identified by GO analysis, the highest degree of tenogenic phenotype maintenance (in the case of hTCs) and tenogenic induction (in the case of hDFs) was attained under combined treatment with aligned PLLA fibres, TGFB2 and MMC. These results align well with the thriving role of microenvironmental cues in cell phenotype governance [41,47,152,153] and underscore the value of multifactorial tendon engineered *in vitro* microenvironments to maintain hTC phenotype and to direct hDFs towards tenogenic lineage. The modulation of the quantitative matrisome of hTCs and hDFs by the employed microenvironmental cues, followed the trend observed in global proteomic analysis. Based on matrisome proteins expression values, samples grouped according to TGFB2-treatment status irrespectively to cellular origins. In agreement with the induction of tendon ECM proteins by TGFB signalling [154] and the enhanced deposition of numerous ECM proteins led by MMC [86,87], +MMC + TGFB2 conditions induced the highest protein levels throughout all matrisome categories; further substantiating the virtues of this approach for tendon tissue engineering [51,154]. Even though tendon-specific ECM proteins were greatly increased under such conditions, their contents were far behind those found in adult tendon tissue, as revealed by principal components analysis. This is in agreement with previous work employing a similarly short culture period [155], which likely leads to the production of tissue engineered constructs with a relatively low ECM-to-cells ratio [156]. Notwithstanding, the present results collectively portray a significant advancement over the state of the art, advocating the approach as a useful technology to produce cell-assembled tendon constructs from hTCs and hDFs [5], which following further maturation and processing, might serve for the development of tendon advanced therapies and *in vitro* systems for disease modelling and drug discovery [157].

## 5 Conclusions

Although tissue engineered medicines hold promise for fully restoring tendon function after injury or disease, their clinical translation is hindered by several issues (e.g. phenotype losses during prolonged *ex vivo* cultures, low ECM deposition even after prolonged *ex vivo* cultures, etc.). Exploiting recent discoveries on the potential of tissue-specific microenvironments to control cell fate, herein, a tendon microenvironment was engineered *in vitro* using aligned PLLA microfibres, TGFB2 and MMC to maintain hTC phenotype and to direct towards tenogenic lineage hDFs. Our approach resulted in tendon-like cellular and supramolecular assemblies, laying the foundations for further studies to develop three-dimensional tendon-like macro-tissues.

## CRediT authorship contribution statement

**Adrian Djalali-Cuevas:** Writing – review & editing, Writing – original draft, Methodology, Investigation, Formal analysis, Data curation. **Mandy Rettel:** Writing – review & editing, Methodology, Investigation, Formal analysis, Data curation. **Frank Stein:** Writing – review & editing, Methodology, Investigation, Formal analysis, Data curation. **Mikhail Savitski:** Writing – review & editing, Methodology, Investigation, Formal analysis, Data curation. **Ioannis Gkiatas:** Writing – review & editing, Resources, Methodology, Investigation. **Anastasios Korompilias:** Writing – review & editing, Resources, Methodology, Investigation. **Ioannis Skoufos:** Writing – review & editing, Supervision, Resources, Project administration, Methodology, Investigation. **Athina Tzora:** Writing – review & editing, Supervision, Resources. **Nikitas Prassinos:** Writing – review & editing, Supervision. **Nikolaos Diakakis:** Writing – review & editing, Supervision. **Dimitrios I. Zeugolis:** Writing – review & editing, Writing – original draft, Visualization, Supervision, Resources, Project administration, Methodology, Investigation, Funding acquisition, Conceptualization.

## Ethics approval and consent to participate

Ethics approval was obtained from the University Hospital of Ioannina. As the project was completed back in 2019, all documents have been destroyed as per local rules and regulations (5 years retention time). We have ethics approvals with other hospitals that are still valid (approval number: RS24-005). A copy of the patient consent form is also provided.

## Data availability statement

Raw and processed date are available upon request from Adrian Djalali-Cuevas.

## Declaration of competing interest

The authors declare that they have no known competing financial interests or personal relationships that could have appeared to influence the work reported in this paper.
