# Supplementary material for: Multifactorial bioengineering in the tendon context to maintain tenocyte phenotype and to direct dermal fibroblasts towards tenogenic lineage
Source: Bioact Mater. 2025 Dec 3;56:743–55. doi: 10.1016/j.bioactmat.2025.11.008 (PMC12747210; doi:10.1016/j.bioactmat.2025.11.008)
Supplement: Multimedia component 12 [file mmc12.docx]

**Supplementary Information**

### Title

Multifactorial bioengineering in the tendon context to maintain tenocyte phenotype and to direct dermal fibroblasts towards tenogenic lineage

# 1. Figures

**Figure S1:** Brightfield microscopy images of Mimetix® aligned poly-L-lactide fibres (The Electrospinning Company, UK), before (**A**, **B**) and after (**C**, **D**) preconditioning with 20 % ethanol, washing with PBS and incubation with basal medium, as per manufacturer’s instructions.


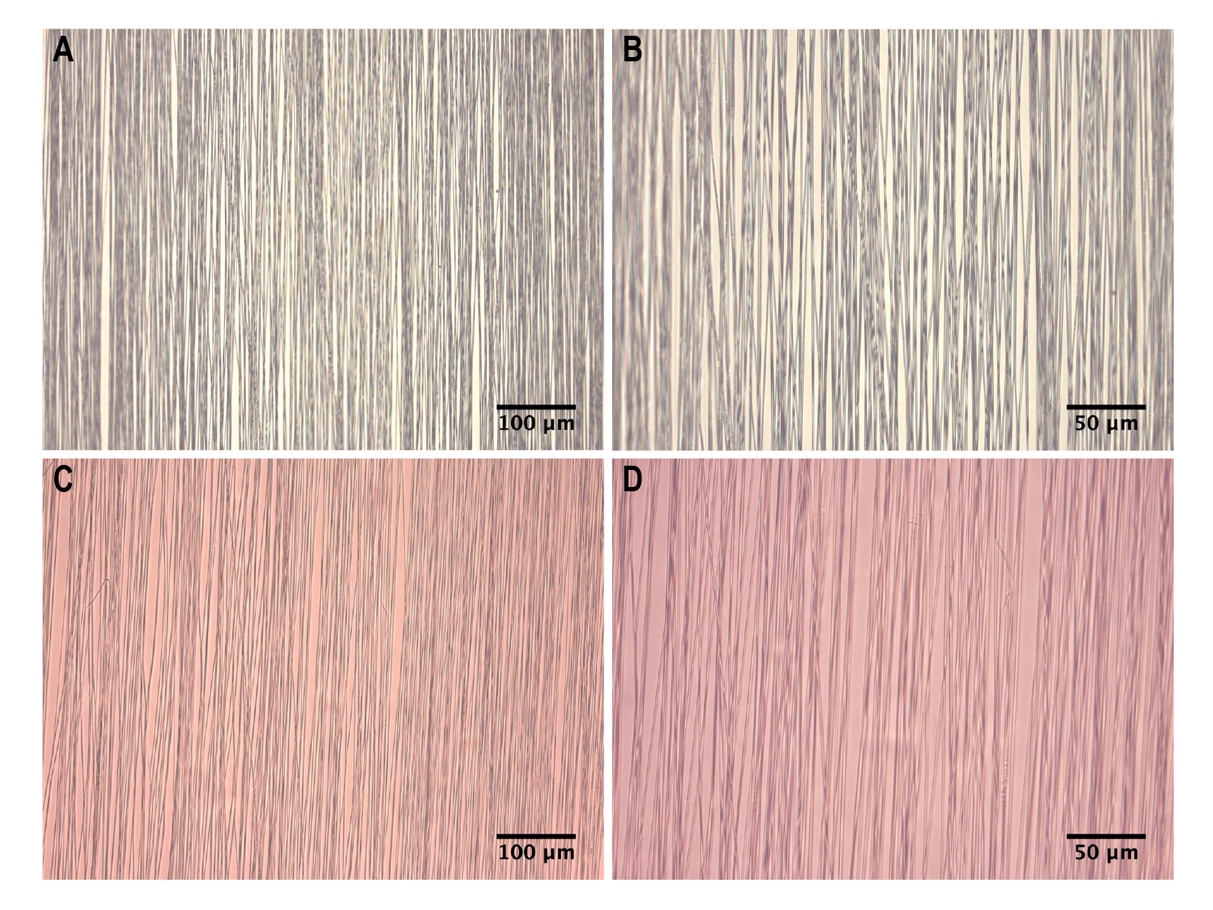


**Figure S2:** Qualitative (**A**) and quantitative (**B**) analysis of cell proliferation of hTCs and hDFs after 4, 7 and 10 days of culture under PLLA, +TGFB2, +MMC and +MMC+TGFB2 experimental conditions. In general, proliferation was higher in hDFs than in hTCs and TGFB2 increased proliferation in both hTCs and hDFs, alone and in combination with MMC; especially after 7 and 10 days of culture. MMC did not affect proliferation at any time point and condition. Proliferation increased as a function of time in culture under all experimental conditions (*p < 0.05 vs respective PLLA group at the same time point; ‡p < 0.05 vs respective +TGFB2 group at the same time point; #p < 0.05 vs. respective +MMC group at the same time point; §p < 0.05 between cell types at the same time point and condition; &p < 0.05 vs. respective condition at day 4). N = 3.


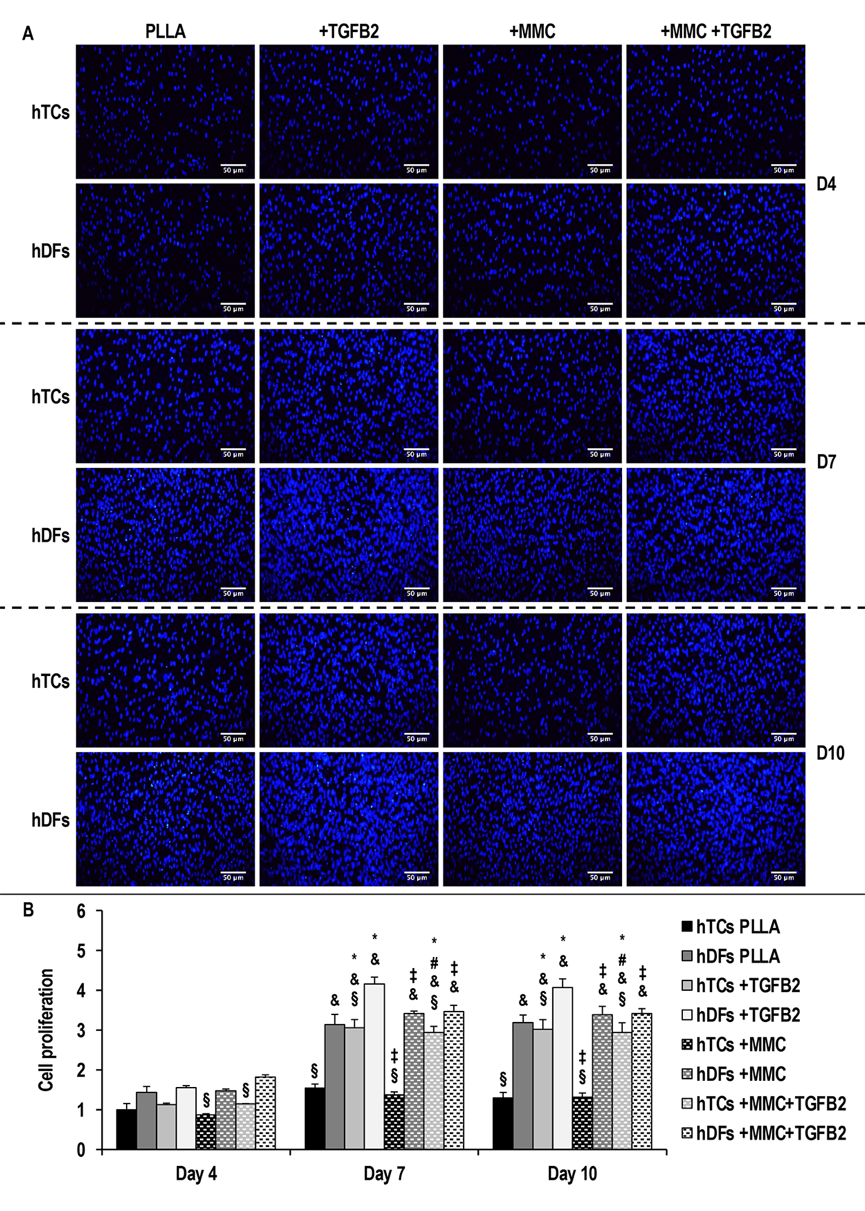


**Figure S3:** Assessment of cell viability of hTCs and hDFs cultured under PLLA, +TGFB2, +MMC and +MMC+TGFB2 conditions for 4, 7 and 10 days, by means of fluorescent staining with calcein-AM and ethidium homodimer. Cell viability was proper and remained unaltered under all experimental conditions and time points. N = 3.


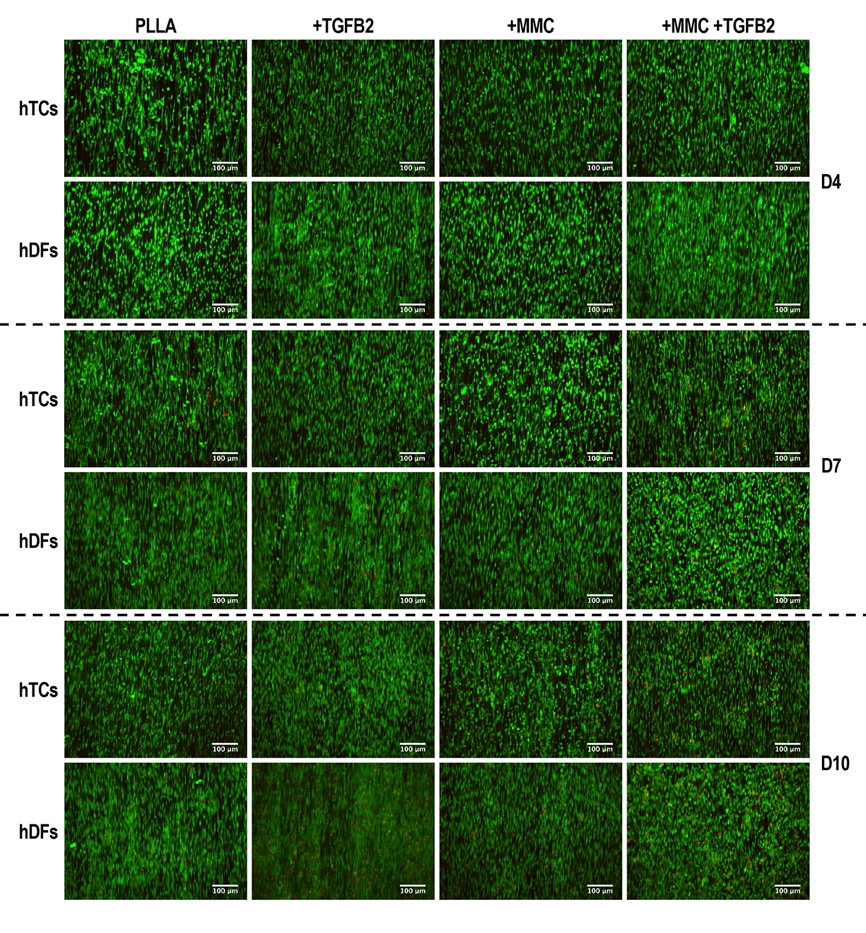


**Figure S4:** Assessment of cell metabolic activity of hTCs and hDFs after 4, 7 and 10 days of culture under PLLA, +TGFB2, +MMC and +MMC+TGFB2 conditions. (*p < 0.05 vs respective PLLA group at the same time point; ‡p < 0.05 vs respective +TGFB2 group at the same time point; #p < 0.05 vs. respective +MMC group at the same time point; §p < 0.05 between cell types at the same time point and condition; &p < 0.05 vs. respective condition at day 4). N = 3.


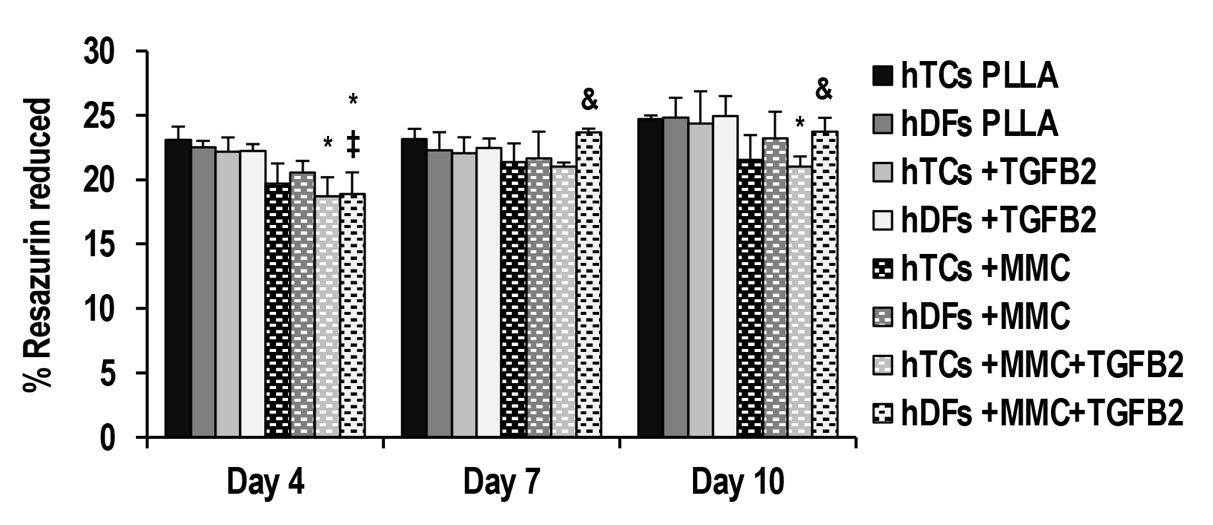


**Figure S5:** Fluorescent immunostaining for COL I in hTCs and hDFs cultured under PLLA, +TGFB2, +MMC, and +MMC+TGFB2 conditions for 4, 7 and 10 days. Deposition of COL I fibres was not detected under PLLA conditions. TGFB2 slightly increased COL I immunostaining in hTCs and hDFs after 7 and 10 days of culture. MMC increased COL I deposition in hTCs and hDFs at all time points. The combination of MMC and TGFB2 induced the highest COL I deposition, after 7 and 10 days of culture. COL I fibres were deposited parallelly to Mimetix® aligned PLLA fibres (arrows). N = 3.


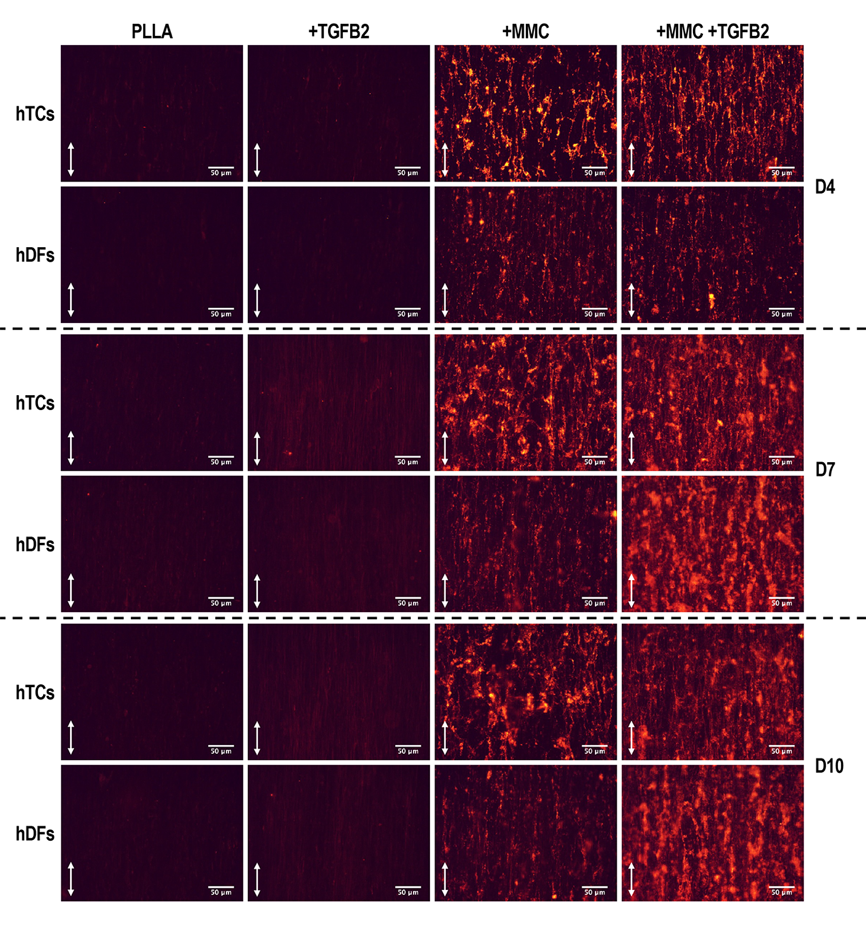


**Figure S6:** Fluorescent immunostaining for COL III in hTCs and hDFs cultured under PLLA, +TGFB2, +MMC, and +MMC+TGFB2 conditions for 4, 7 and 10 days. Deposition of COL III fibres was low under PLLA conditions. TGFB2 slightly increased COL III immunostaining in hTCs and hDFs after 7 and 10 days of culture. MMC increased COL III deposition in hTCs and hDFs at all time points. The combination of MMC and TGFB2 induced the highest COL III deposition, after 7 and 10 days of culture. COL III fibres were deposited parallelly to Mimetix® aligned PLLA fibres (arrows). N = 3.


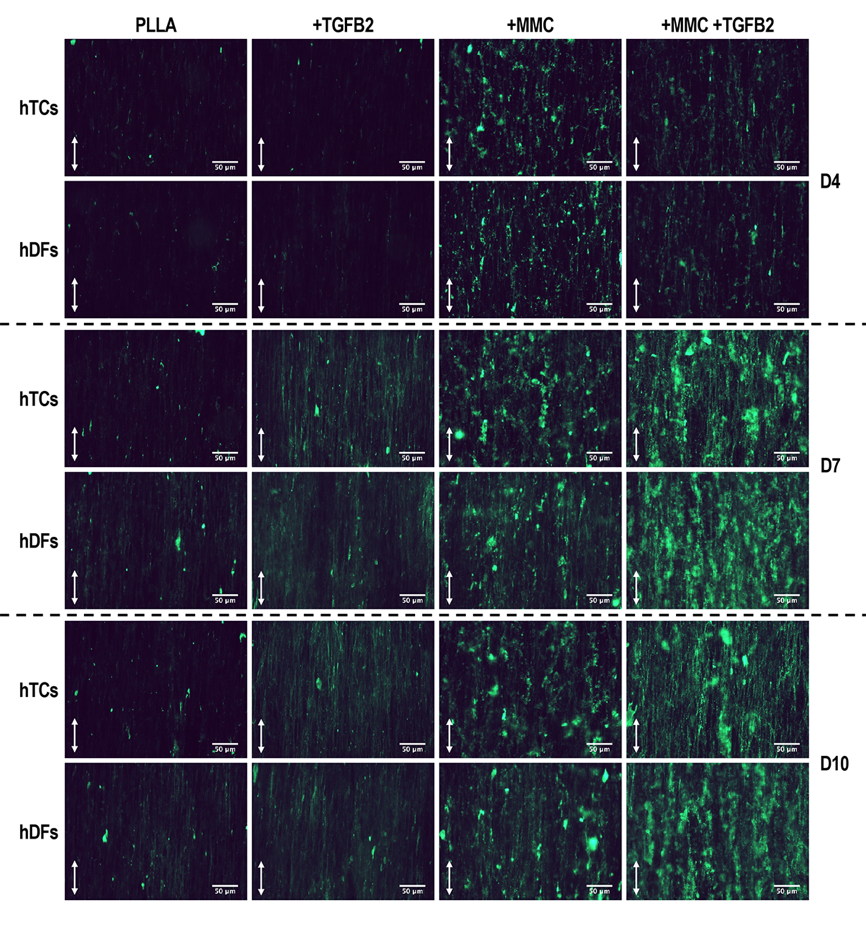


**Figure S7:** Fluorescent immunostaining for COL IV in hTCs and hDFs cultured under PLLA, +TGFB2, +MMC, and +MMC+TGFB2 conditions for 4, 7 and 10 days. Deposition of COL IV fibres remained low under PLLA conditions. TGFB2 slightly increased COL IV immunostaining in hTCs and hDFs after 7 and 10 days of culture. MMC increased COL IV deposition in hTCs and hDFs at all time points. The combination of MMC and TGFB2 induced the highest COL IV deposition, after 7 and 10 days of culture. COL IV fibres were deposited parallelly to Mimetix® aligned PLLA fibres (arrows). N = 3.


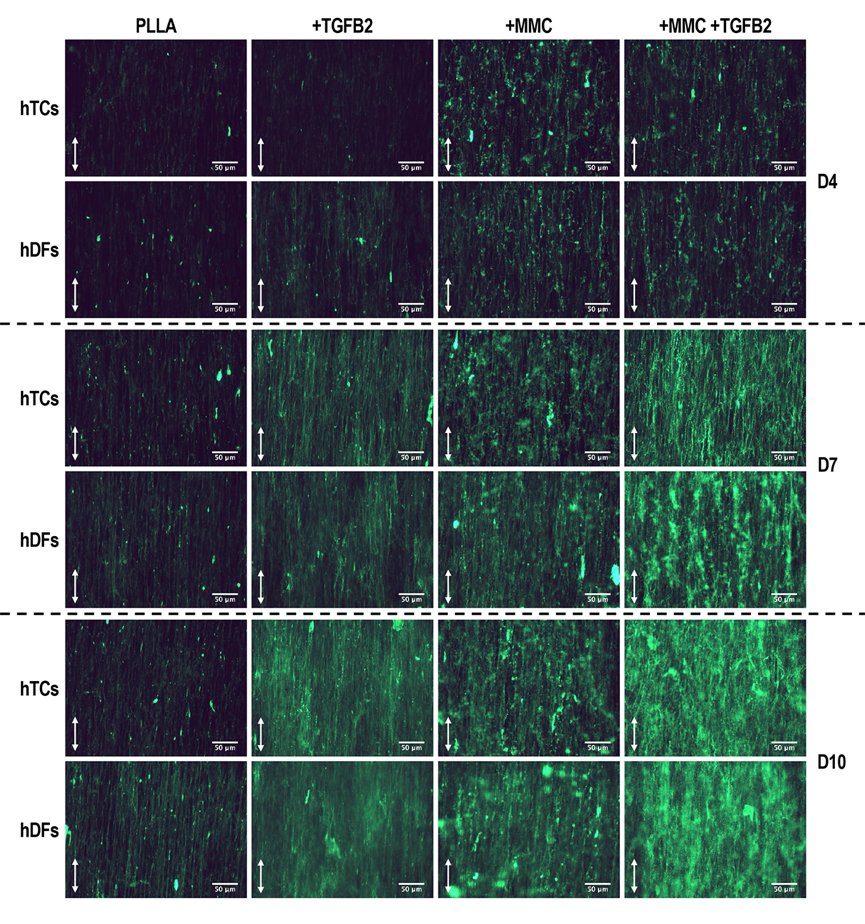


**Figure S8:** Fluorescent immunostaining for COL V in hTCs and hDFs cultured under PLLA, +TGFB2, +MMC, and +MMC+TGFB2 conditions for 4, 7 and 10 days. The lowest deposition of COL V fibres was observed under PLLA conditions. TGFB2 increased COL V deposition in hTCs and hDFs, especially after 10 days of culture. Deposition of COL V was increased by MMC at all time points in hTCs and hDFs, and at higher levels in the latter. The combination of MMC and TGFB2 induced the highest COL V deposition, after 7 and 10 days of culture. COL IV fibres were deposited parallelly to Mimetix® aligned PLLA fibres (arrows). N = 3.


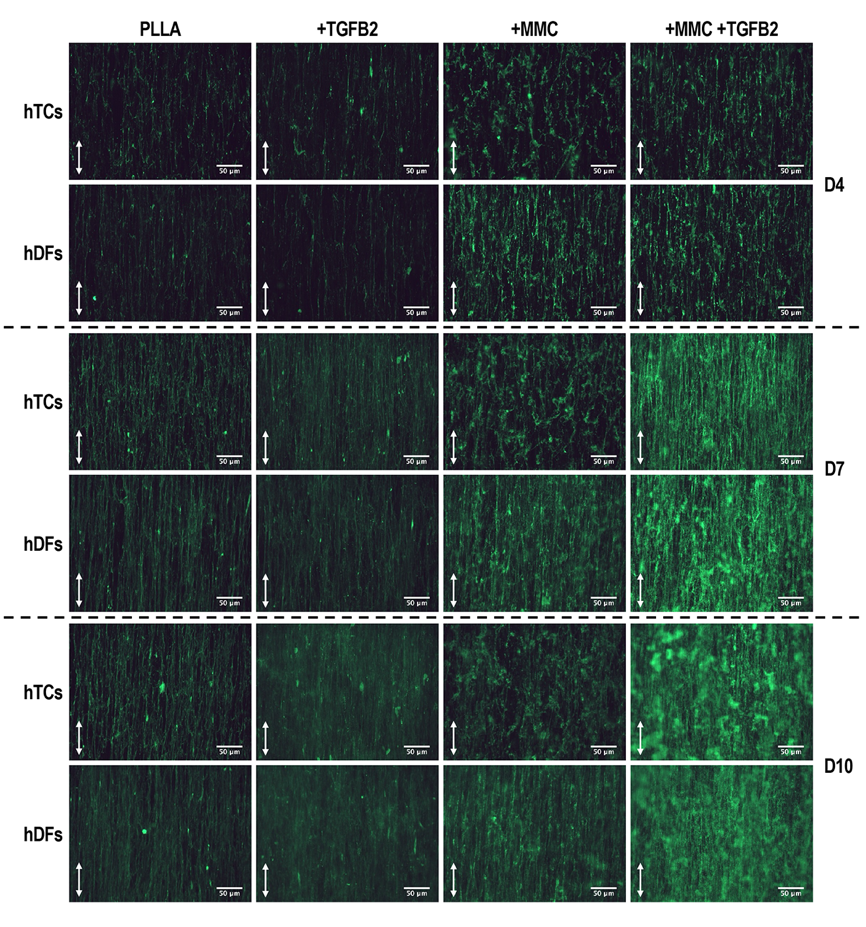


**Figure S9:** Fluorescent immunostaining for COL VI in hTCs and hDFs cultured under PLLA, +TGFB2, +MMC, and +MMC+TGFB2 conditions for 4, 7 and 10 days. Deposition of COL VI fibres was observed under under all experimental conditions at all time points. In general, TGFB2 decreased the deposition of COL VI fibres, alone or in combination with MMC. MMC did not increase the deposition of COL VI fibres at any time point or condition. COL IV fibres were deposited parallelly to Mimetix® aligned PLLA fibres (arrows). N = 3.


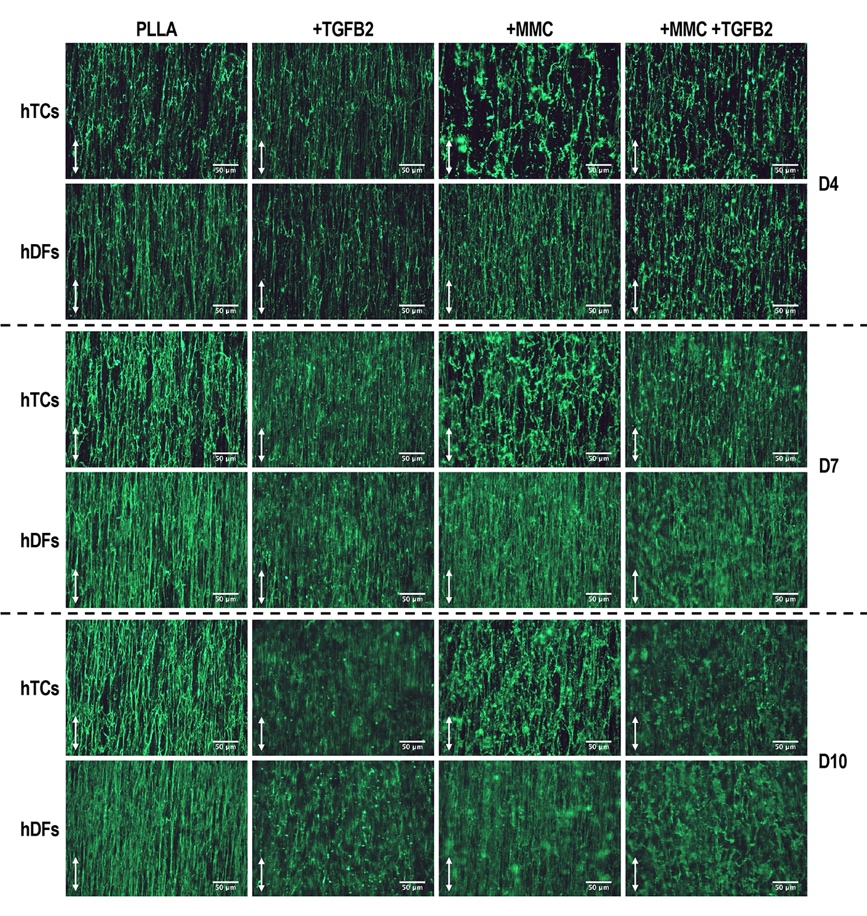


**Figure S10:** Immunofluorescent staining for TNMD in hTCs and hDFs cultured under PLLA, +TGFB2, +MMC, and +MMC+TGFB2 conditions for 4, 7 and 10 days. TNMD expression was detected in hTCs and hDFs under all experimental conditions at all time points. N = 3.


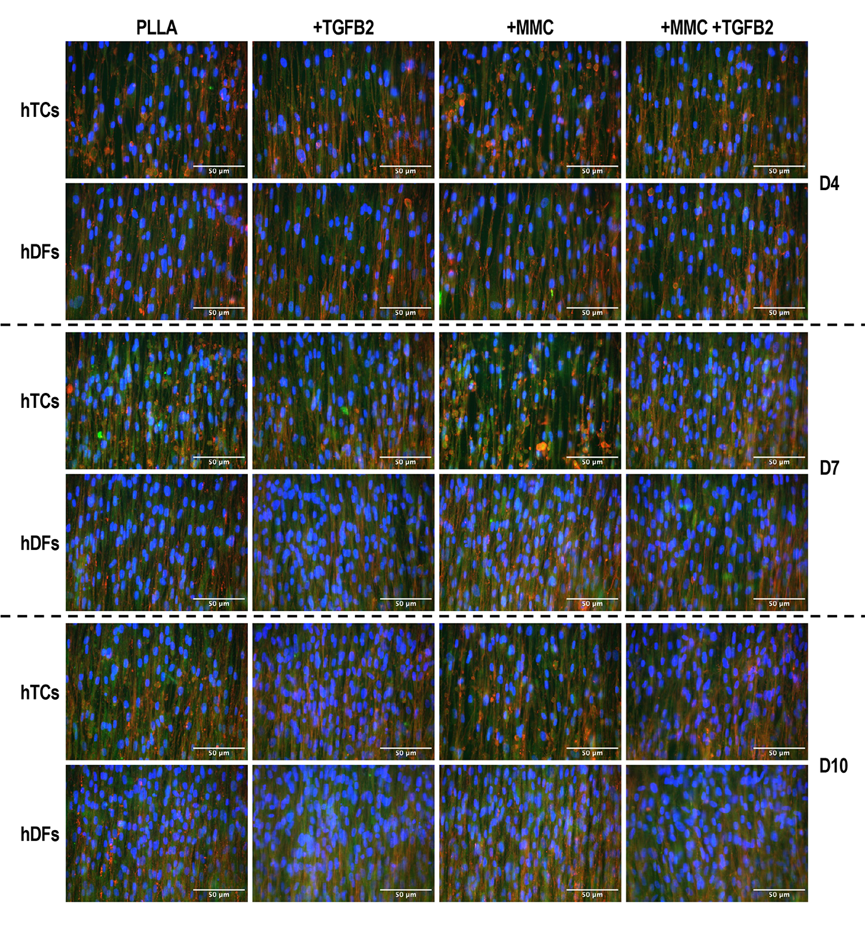


**Figure S11:** Immunofluorescent staining for aSMA in hTCs and hDFs cultured under PLLA, +TGFB2, +MMC, and +MMC+TGFB2 conditions for 4, 7 and 10 days. Myofibroblasts (arrowheads) were detected in hTCs and hDFs cultured under +TGFB2 and +MMC+TGFB2 conditions at all time points, and under +MMC conditions at day 4. The proportion of myofibroblast cells remained marginal in every case. N = 3.


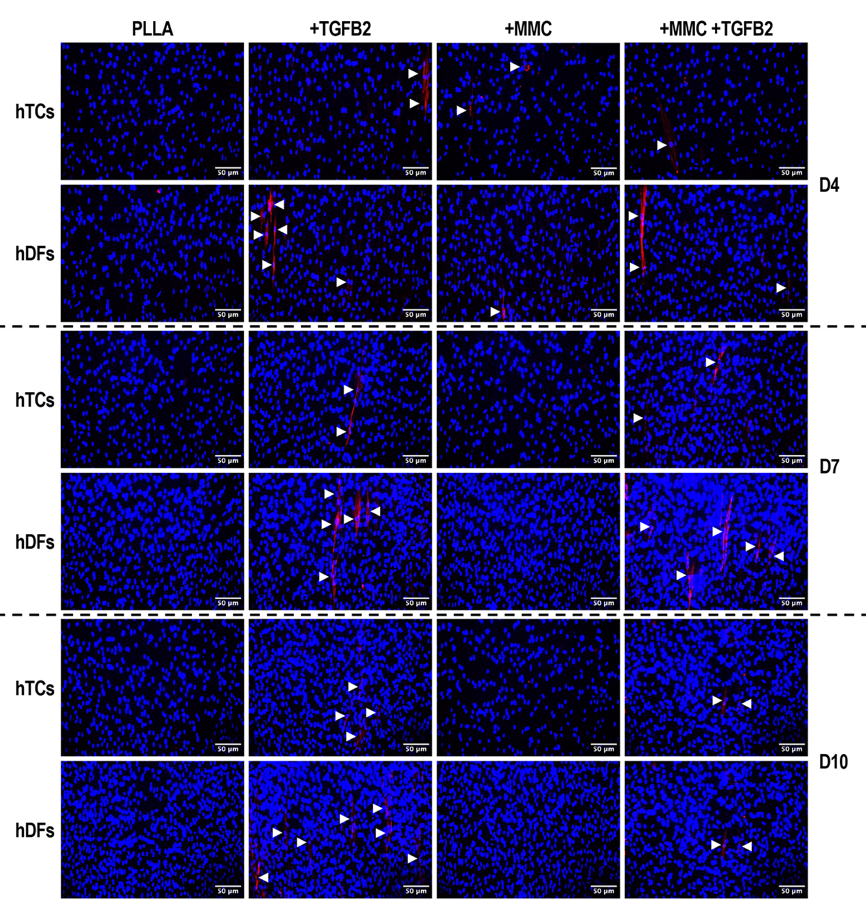


**Figure S12:** Analysis of cell morphology of hTCs and hDFs by means of F-actin fluorescent staining. Both cell types possessed a similar spindle-shaped morphology that remained unaltered as a function of time in culture or experimental treatment. N = 3.


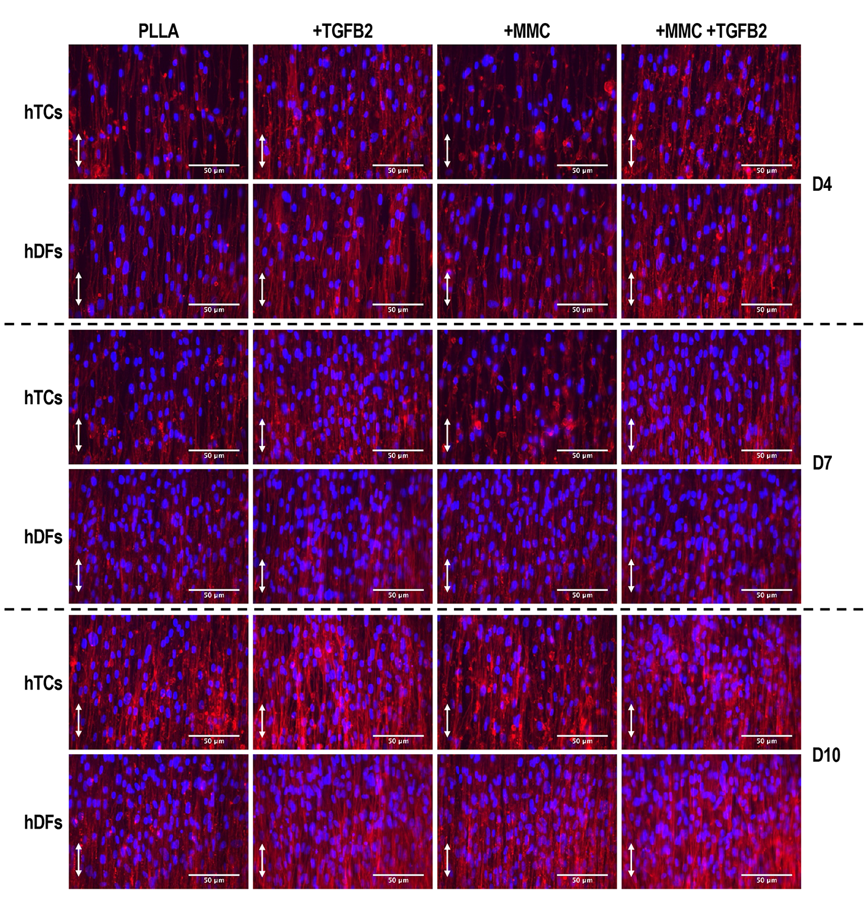


**Figure S13:** Assessment of nuclear orientation in hTCs and hDFs after 4, 7 and 10 days in culture under PLLA, +TGFB2, +MMC and +MMC+TGFB2 conditions. Under all experimental conditions and time points, the major axis of the cell nuclei was predominantly aligned at 90º, in parallel to the Mimetix® aligned PLLA fibres. N = 3.


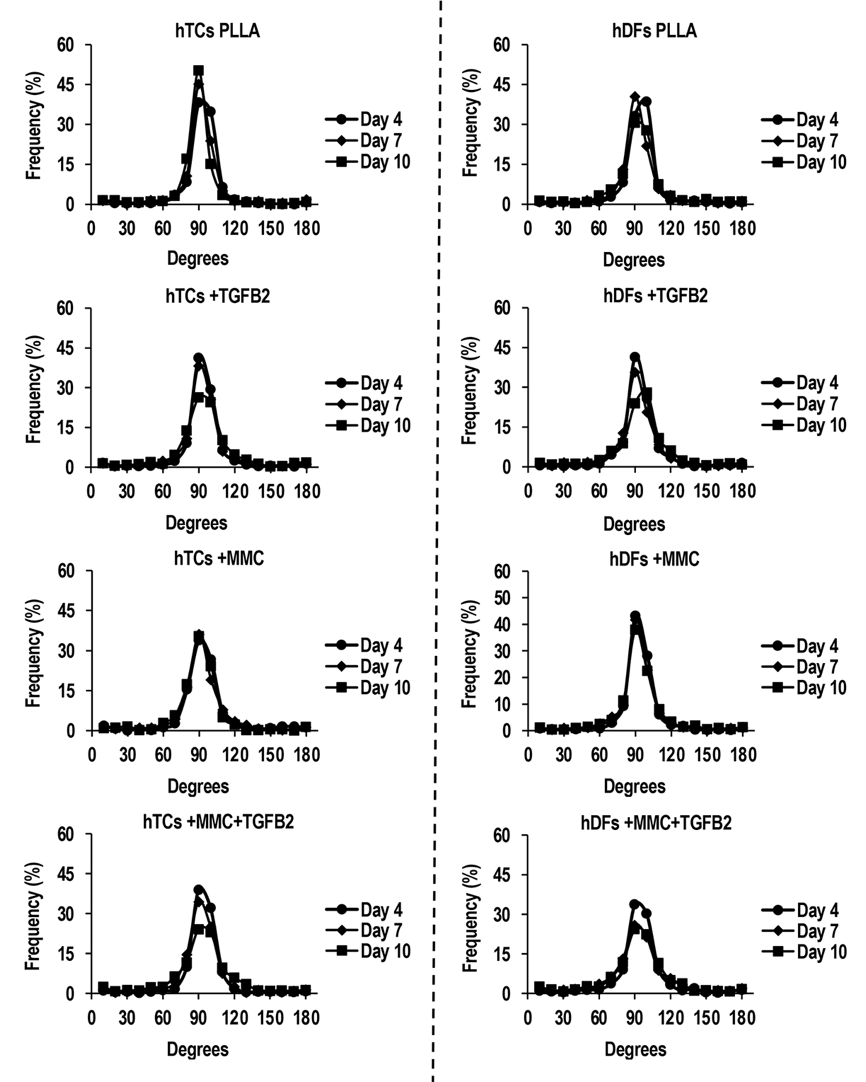


**Figure S14:** Assessment of the main orientation of cytoskeletal (F-Actin) fibres in hTCs and hDFs cultured under PLLA, +TGFB2, +MMC and +MMC+TGFB2 conditions for 4, 7 and 10 days. The main orientation of the cytoskeletal fibres was close to 90º, aligned in a parallel fashion to the Mimetix® aligned PLLA fibres, under all experimental conditions and time points. No significant (p > 0.05) differences were found between experimental conditions and time points. (*p < 0.05 vs respective PLLA group at the same time point; ‡p < 0.05 vs respective +TGFB2 group at the same time point; #p < 0.05 vs. respective +MMC group at the same time point; §p < 0.05 between cell types at the same time point and condition; &p < 0.05 vs. respective condition at day 4). N = 3.


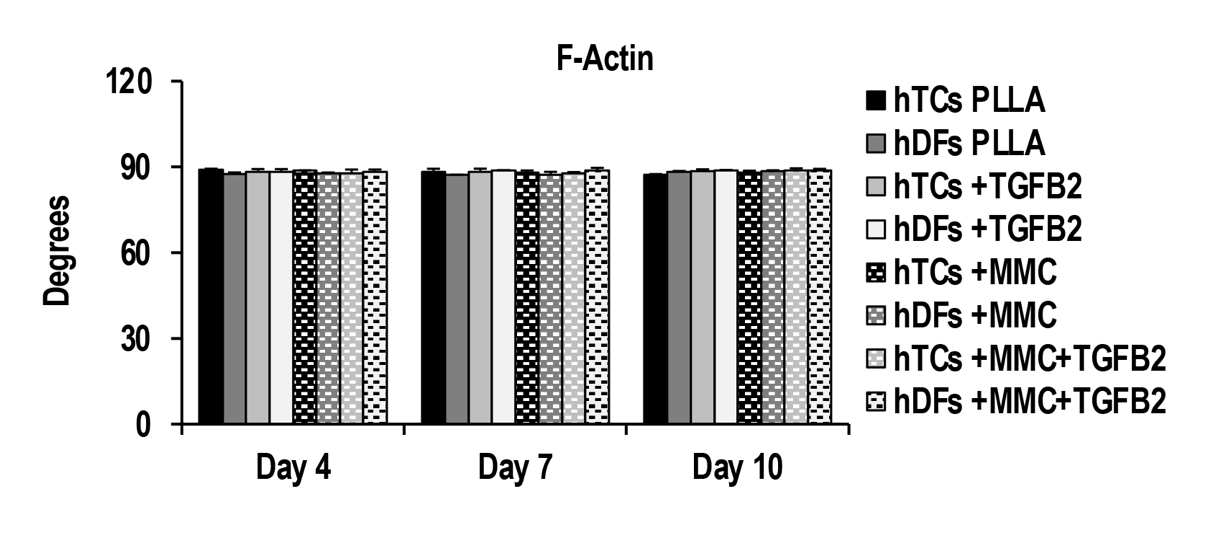


**Figure S15:** Assessment of the main orientation of deposited COL I, III, IV, V and VI fibres in hTCs and hDFs cultured under PLLA, +TGFB2, +MMC and +MMC+TGFB2 conditions for 4, 7 and 10 days. The main orientation of all collagen fibres was close to 90º, aligned in a parallel fashion to the Mimetix® aligned PLLA fibres, under all experimental conditions and time points. No significant (p > 0.05) differences were found between experimental conditions and time points. (*p < 0.05 vs respective PLLA group at the same time point; ‡p < 0.05 vs respective +TGFB2 group at the same time point; #p < 0.05 vs. respective +MMC group at the same time point; §p < 0.05 between cell types at the same time point and condition; &p < 0.05 vs. respective condition at day 4). N = 3.


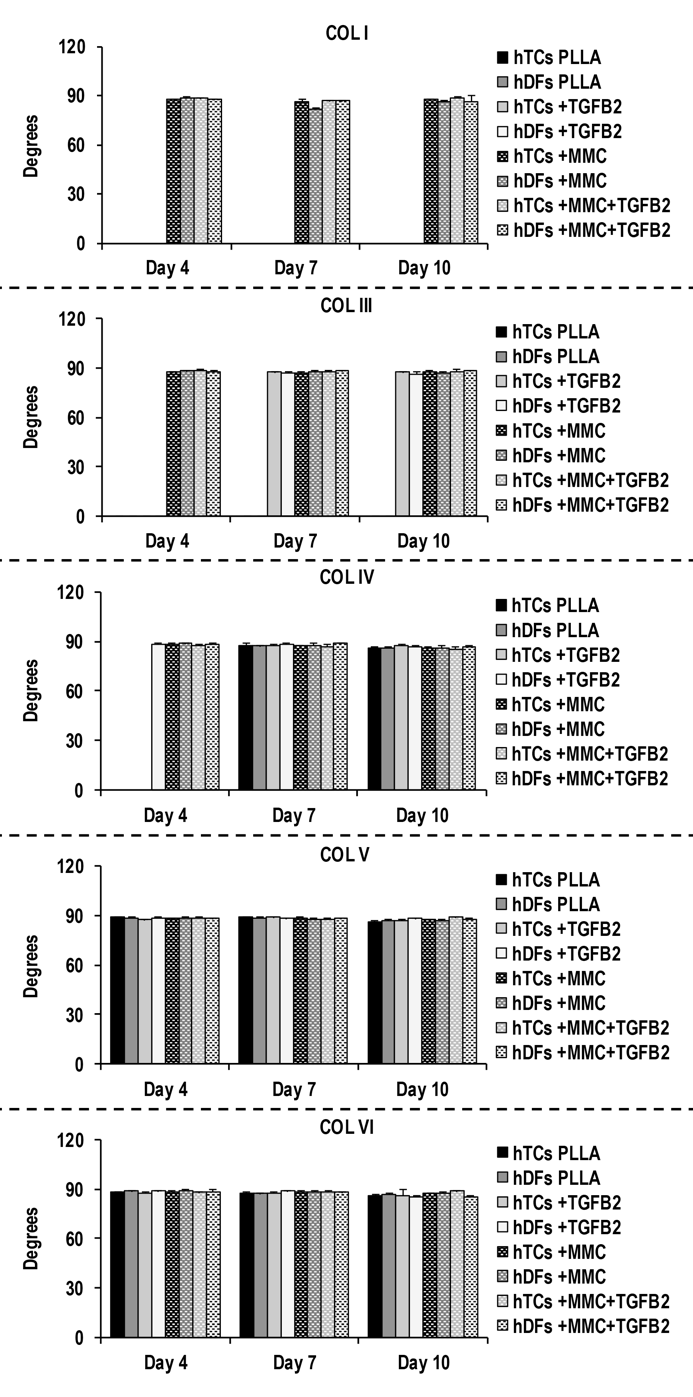


# 2. Tables

**Table S1:** Donor details and characteristics.

| **Tissue / Cell type** | **Sex** | **Age** | **Race** |
| --- | --- | --- | --- |
| Tendon / hTCs | Female | 16 | Caucasian |
| Tendon / hTCs | Male | 18 | Caucasian |
| Tendon / hTCs | Female | 50 | Caucasian |
| hDFs | Female | 38 | Caucasian |
| hDFs | Male | 28 | African American |
| hDFs | Female | 40 | Asian |

**Table S2:** List of antibodies used and their characteristics.

| **Antigen detected** | **Host species** | **Clonality** | **Dilution factor** | **Product number** | **Manufacturer** |
| --- | --- | --- | --- | --- | --- |
| TNMD | Rabbit | Polyclonal | 1:200 | ab203676 | Abcam, UK |
| aSMA | Mouse | Monoclonal | 1:400 | A2547 | Sigma-Aldrich, GR |
| COL I | Mouse | Monoclonal | 1:200 | ab90395 | Abcam, UK |
| COL III | Rabbit | Polyclonal | 1:200 | ab7778 | Abcam, UK |
| COL IV | Rabbit | Polyclonal | 1:200 | ab6586 | Abcam, UK |
| COL V | Rabbit | Polyclonal | 1:200 | ab7046 | Abcam, UK |
| COL VI | Rabbit | Polyclonal | 1:200 | ab6588 | Abcam, UK |
| Mouse IgG | Goat | Polyclonal | 1:400 | A-32727 | Invitrogen, IE |
| Rabbit IgG | Goat | Polyclonal | 1:400 | A-32731 | Invitrogen, IE |

**Table S3:** Immunofluorescence intensity analysis at day 4, day 7 and day 10 of PLLA Vs +TGFB2, PLLA Vs +MMC, PLLA Vs +MMC+TGFB2, +TGFB2 Vs +MMC, +TGFB2 Vs +MMC+TGFB2 and +MMC Vs +MMC+TGFB2 for hTCs and hDFs. Green background indicates significantly (p < 0.05) higher intensity in the second group. Red background indicates significantly (p < 0.05) lower intensity in the second group. White background indicates no significant (p > 0.05) difference. N = 3.

|  | **PLLA Vs +TGFB2** | **PLLA Vs +MMC** | **PLLA Vs +MMC +TGFB2** | **+TGFB2 Vs +MMC** | **+TGFB2 Vs +MMC + TGFB2** | **+MMC Vs +MMC+TGFB2** | **PLLA Vs +TGFB2** | **PLLA Vs +MMC** | **PLLA Vs +MMC +TGFB2** | **+TGFB2 Vs +MMC** | **+TGFB2 Vs +MMC + TGFB2** | **+MMC Vs +MMC+TGFB2** | **PLLA Vs +TGFB2** | **PLLA Vs +MMC** | **PLLA Vs +MMC +TGFB2** | **+TGFB2 Vs +MMC** | **+TGFB2 Vs +MMC + TGFB2** | **+MMC Vs +MMC+TGFB2** |
| --- | --- | --- | --- | --- | --- | --- | --- | --- | --- | --- | --- | --- | --- | --- | --- | --- | --- | --- |
|  | **hTCs Day 4** | | | | | | **hTCs Day 7** | | | | | | **hTCs Day 10** | | | | | |
| **Col I** | p > 0.05 | p < 0.05 | p < 0.05 | p < 0.05 | p < 0.05 | p > 0.05 | p < 0.05 | p < 0.05 | p < 0.05 | p < 0.05 | p < 0.05 | p < 0.05 | p < 0.05 | p < 0.05 | p < 0.05 | p < 0.05 | p < 0.05 | p < 0.05 |
| **Col III** | p > 0.05 | p < 0.05 | p < 0.05 | p < 0.05 | p < 0.05 | p > 0.05 | p < 0.05 | p < 0.05 | p < 0.05 | p < 0.05 | p < 0.05 | p < 0.05 | p < 0.05 | p < 0.05 | p < 0.05 | p > 0.05 | p < 0.05 | p < 0.05 |
| **Col IV** | p > 0.05 | p < 0.05 | p < 0.05 | p < 0.05 | p < 0.05 | p > 0.05 | p < 0.05 | p < 0.05 | p < 0.05 | p > 0.05 | p < 0.05 | p < 0.05 | p < 0.05 | p < 0.05 | p < 0.05 | p > 0.05 | p < 0.05 | p < 0.05 |
| **Col V** | p > 0.05 | p < 0.05 | p < 0.05 | p < 0.05 | p < 0.05 | p > 0.05 | p < 0.05 | p > 0.05 | p < 0.05 | p > 0.05 | p < 0.05 | p < 0.05 | p < 0.05 | p < 0.05 | p < 0.05 | p < 0.05 | p < 0.05 | p < 0.05 |
| **Col VI** | p > 0.05 | p > 0.05 | p > 0.05 | p > 0.05 | p > 0.05 | p > 0.05 | p < 0.05 | p > 0.05 | p < 0.05 | p > 0.05 | p > 0.05 | p > 0.05 | p < 0.05 | p < 0.05 | p < 0.05 | p < 0.05 | p > 0.05 | p < 0.05 |
| **COL I / COL III** | N/A | N/A | N/A | N/A | N/A | p > 0.05 | N/A | N/A | N/A | p < 0.05 | p < 0.05 | p > 0.05 | N/A | N/A | N/A | p < 0.05 | p < 0.05 | p > 0.05 |
| **TNMD** | p > 0.05 | p > 0.05 | p > 0.05 | p > 0.05 | p > 0.05 | p > 0.05 | p > 0.05 | p > 0.05 | p > 0.05 | p > 0.05 | p > 0.05 | p > 0.05 | p < 0.05 | p > 0.05 | p < 0.05 | p > 0.05 | p > 0.05 | p > 0.05 |
| **aSMA** | p > 0.05 | p > 0.05 | p > 0.05 | p > 0.05 | p > 0.05 | p > 0.05 | p > 0.05 | p > 0.05 | p > 0.05 | p > 0.05 | p > 0.05 | p > 0.05 | p > 0.05 | p > 0.05 | p > 0.05 | p > 0.05 | p > 0.05 | p > 0.05 |
|  | **hDFs Day 4** | | | | | | **hDFs Day 7** | | | | | | **hDFs Day 10** | | | | | |
| **Col I** | p > 0.05 | p < 0.05 | p < 0.05 | p < 0.05 | p < 0.05 | p > 0.05 | p < 0.05 | p < 0.05 | p < 0.05 | p > 0.05 | p < 0.05 | p < 0.05 | p < 0.05 | p < 0.05 | p < 0.05 | p < 0.05 | p < 0.05 | p < 0.05 |
| **Col III** | p > 0.05 | p < 0.05 | p < 0.05 | p < 0.05 | p < 0.05 | p > 0.05 | p < 0.05 | p < 0.05 | p < 0.05 | p > 0.05 | p < 0.05 | p < 0.05 | p < 0.05 | p < 0.05 | p < 0.05 | p < 0.05 | p < 0.05 | p < 0.05 |
| **Col IV** | p < 0.05 | p < 0.05 | p < 0.05 | p < 0.05 | p > 0.05 | p > 0.05 | p < 0.05 | p < 0.05 | p < 0.05 | p < 0.05 | p < 0.05 | p < 0.05 | p < 0.05 | p < 0.05 | p < 0.05 | p > 0.05 | p < 0.05 | p < 0.05 |
| **Col V** | p > 0.05 | p < 0.05 | p < 0.05 | p < 0.05 | p < 0.05 | p > 0.05 | p > 0.05 | p < 0.05 | p < 0.05 | p < 0.05 | p < 0.05 | p < 0.05 | p < 0.05 | p < 0.05 | p < 0.05 | p < 0.05 | p < 0.05 | p < 0.05 |
| **Col VI** | p < 0.05 | p > 0.05 | p > 0.05 | p < 0.05 | p < 0.05 | p > 0.05 | p < 0.05 | p > 0.05 | p < 0.05 | p < 0.05 | p > 0.05 | p > 0.05 | p < 0.05 | p < 0.05 | p < 0.05 | p < 0.05 | p > 0.05 | p > 0.05 |
| **COL I / COL III** | N/A | N/A | N/A | N/A | N/A | p > 0.05 | N/A | N/A | N/A | p < 0.05 | p < 0.05 | p < 0.05 | N/A | N/A | N/A | p < 0.05 | p < 0.05 | p < 0.05 |
| **TNMD** | p > 0.05 | p > 0.05 | p > 0.05 | p > 0.05 | p > 0.05 | p > 0.05 | p > 0.05 | p > 0.05 | p > 0.05 | p > 0.05 | p > 0.05 | p > 0.05 | p > 0.05 | p > 0.05 | p > 0.05 | p > 0.05 | p > 0.05 | p > 0.05 |
| **aSMA** | p > 0.05 | p > 0.05 | p > 0.05 | p > 0.05 | p > 0.05 | p > 0.05 | p > 0.05 | p > 0.05 | p > 0.05 | p > 0.05 | p > 0.05 | p > 0.05 | p > 0.05 | p > 0.05 | p > 0.05 | p > 0.05 | p > 0.05 | p > 0.05 |

**Table S4:** Immunofluorescence intensity analysis at day 4, day 7 and day 10 of hTCs Vs hDFs cultured under PLLA, +TGFB2, +MMC and +MMC+TGFB2 conditions. Green background indicates significantly (p < 0.05) higher intensity in hDFs. Red background indicates significantly (p < 0.05) higher intensity in hTCs. White background indicates no significant (p > 0.05) difference. N = 3.

|  | **PLLA** | **+TGFB2** | **+MMC** | **+MMC +TGFB2** | **PLLA** | **+TGFB2** | **+MMC** | **+MMC +TGFB2** | **PLLA** | **+TGFB2** | **+MMC** | **+MMC +TGFB2** |
| --- | --- | --- | --- | --- | --- | --- | --- | --- | --- | --- | --- | --- |
|  | **Day 4** | | | | **Day 7** | | | | **Day 10** | | | |
| **Col I** | p > 0.05 | p > 0.05 | p < 0.05 | p < 0.05 | p > 0.05 | p > 0.05 | p < 0.05 | p < 0.05 | p > 0.05 | p > 0.05 | p < 0.05 | p > 0.05 |
| **Col III** | p > 0.05 | p > 0.05 | p > 0.05 | p > 0.05 | p > 0.05 | p > 0.05 | p > 0.05 | p > 0.05 | p < 0.05 | p > 0.05 | p > 0.05 | p > 0.05 |
| **Col IV** | p > 0.05 | p < 0.05 | p > 0.05 | p > 0.05 | p > 0.05 | p > 0.05 | p > 0.05 | p > 0.05 | p < 0.05 | p > 0.05 | p > 0.05 | p > 0.05 |
| **Col V** | p > 0.05 | p > 0.05 | p < 0.05 | p < 0.05 | p > 0.05 | p < 0.05 | p < 0.05 | p < 0.05 | p < 0.05 | p > 0.05 | p < 0.05 | p > 0.05 |
| **Col VI** | p > 0.05 | p > 0.05 | p > 0.05 | p > 0.05 | p > 0.05 | p > 0.05 | p > 0.05 | p > 0.05 | p > 0.05 | p > 0.05 | p > 0.05 | p > 0.05 |
| **COL I / COL III** | N/A | N/A | p < 0.05 | p < 0.05 | N/A | p < 0.05 | p < 0.05 | p > 0.05 | N/A | p < 0.05 | p < 0.05 | p > 0.05 |
| **TNMD** | p > 0.05 | p > 0.05 | p > 0.05 | p > 0.05 | p > 0.05 | p > 0.05 | p > 0.05 | p > 0.05 | p < 0.05 | p > 0.05 | p > 0.05 | p > 0.05 |
| **aSMA** | p > 0.05 | p > 0.05 | p > 0.05 | p > 0.05 | p > 0.05 | p > 0.05 | p > 0.05 | p > 0.05 | p > 0.05 | p > 0.05 | p > 0.05 | p > 0.05 |

**Table S5:** Immunofluorescence intensity analysis of day 4 Vs day 7, day 7 Vs day 10 and day 4 Vs day 10 of hTCs and hDFs cultured under PLLA, +TGFB2, +MMC and +TGFB2 +MMC conditions. Green background indicates significantly (p < 0.05) higher intensity at the second time point. Red background indicates significantly (p < 0.05) lower intensity at the second time point. White background indicates no significant (p > 0.05) difference. N = 3.

|  | **Day 4 Vs Day 7** | **Day 7 Vs Day 10** | **Day 4 Vs Day 10** | **Day 4 Vs Day 7** | **Day 7 Vs Day 10** | **Day 4 Vs Day 10** | **Day 4 Vs Day 7** | **Day 7 Vs Day 10** | **Day 4 Vs Day 10** | **Day 4 Vs Day 7** | **Day 7 Vs Day 10** | **Day 4 Vs Day 10** |
| --- | --- | --- | --- | --- | --- | --- | --- | --- | --- | --- | --- | --- |
|  | **hTCs PLLA** | | | **hTCs +TGFB2** | | | **hTCs +MMC** | | | **hTCs +MMC+TGFB2** | | |
| **Col I** | p > 0.05 | p > 0.05 | p > 0.05 | p < 0.05 | p > 0.05 | p < 0.05 | p > 0.05 | p > 0.05 | p > 0.05 | p < 0.05 | p > 0.05 | p < 0.05 |
| **Col III** | p > 0.05 | p > 0.05 | p > 0.05 | p < 0.05 | p > 0.05 | p < 0.05 | p < 0.05 | p > 0.05 | p < 0.05 | p < 0.05 | p > 0.05 | p < 0.05 |
| **Col IV** | p < 0.05 | p < 0.05 | p < 0.05 | p < 0.05 | p < 0.05 | p < 0.05 | p < 0.05 | p < 0.05 | p < 0.05 | p < 0.05 | p < 0.05 | p < 0.05 |
| **Col V** | p < 0.05 | p > 0.05 | p < 0.05 | p < 0.05 | p < 0.05 | p < 0.05 | p < 0.05 | p > 0.05 | p < 0.05 | p < 0.05 | p < 0.05 | p < 0.05 |
| **Col VI** | p < 0.05 | p > 0.05 | p < 0.05 | p < 0.05 | p < 0.05 | p > 0.05 | p < 0.05 | p > 0.05 | p < 0.05 | p < 0.05 | p > 0.05 | p > 0.05 |
| **COL I / COL III** | N/A | N/A | N/A | N/A | p > 0.05 | N/A | p > 0.05 | p > 0.05 | p < 0.05 | p < 0.05 | p > 0.05 | p < 0.05 |
| **TNMD** | p > 0.05 | p > 0.05 | p > 0.05 | p > 0.05 | p > 0.05 | p > 0.05 | p > 0.05 | p > 0.05 | p > 0.05 | p > 0.05 | p > 0.05 | p > 0.05 |
| **aSMA** | p > 0.05 | p > 0.05 | p > 0.05 | p > 0.05 | p > 0.05 | p > 0.05 | p > 0.05 | p > 0.05 | p > 0.05 | p > 0.05 | p > 0.05 | p > 0.05 |
|  | **hDFs PLLA** | | | **hDFs +TGFB2** | | | **hDFs +MMC** | | | **hDFs +MMC +TGFB2** | | |
| **Col I** | p > 0.05 | p > 0.05 | p > 0.05 | p < 0.05 | p > 0.05 | p < 0.05 | p > 0.05 | p > 0.05 | p > 0.05 | p < 0.05 | p > 0.05 | p < 0.05 |
| **Col III** | p < 0.05 | p > 0.05 | p < 0.05 | p < 0.05 | p > 0.05 | p < 0.05 | p < 0.05 | p > 0.05 | p < 0.05 | p < 0.05 | p > 0.05 | p < 0.05 |
| **Col IV** | p < 0.05 | p < 0.05 | p < 0.05 | p < 0.05 | p < 0.05 | p < 0.05 | p < 0.05 | p < 0.05 | p < 0.05 | p < 0.05 | p < 0.05 | p < 0.05 |
| **Col V** | p < 0.05 | p > 0.05 | p < 0.05 | p < 0.05 | p < 0.05 | p < 0.05 | p < 0.05 | p > 0.05 | p < 0.05 | p < 0.05 | p > 0.05 | p < 0.05 |
| **Col VI** | p < 0.05 | p > 0.05 | p < 0.05 | p < 0.05 | p > 0.05 | p < 0.05 | p < 0.05 | p < 0.05 | p < 0.05 | p < 0.05 | p > 0.05 | p < 0.05 |
| **COL I / COL III** | N/A | N/A | N/A | N/A | p > 0.05 | N/A | p < 0.05 | p >0.05 | p < 0.05 | p >0.05 | p >0.05 | p >0.05 |
| **TNMD** | p > 0.05 | p > 0.05 | p > 0.05 | p > 0.05 | p > 0.05 | p > 0.05 | p > 0.05 | p > 0.05 | p > 0.05 | p > 0.05 | p > 0.05 | p > 0.05 |
| **aSMA** | p > 0.05 | p > 0.05 | p > 0.05 | p > 0.05 | p > 0.05 | p > 0.05 | p > 0.05 | p > 0.05 | p > 0.05 | p > 0.05 | p > 0.05 | p > 0.05 |

**Table S6:** GO annotation of core enrichment proteins in hTCs PLLA. N = 3.

| **Category** | **Sub-category** | **Proteins** | **Fold-enrichment** | **FDR** |
| --- | --- | --- | --- | --- |
| Cellular component | Collagen-containing ECM | 15 | 15.29 | 1.06E-10 |
|  | Extracellular matrix | 16 | 9.6 | 6.17E-09 |
|  | External encapsulating structure | 16 | 9.6 | 4.11E-09 |
|  | Extracellular space | 21 | 2.87 | 1.39E-03 |
|  | Extracellular region | 21 | 2.73 | 2.39E-03 |
|  | Cell periphery | 43 | 2.66 | 1.17E-07 |

**Table S7:** GO annotation of core enrichment proteins in hTCs +TGFB2. N = 3.

| **Category** | **Sub-category** | **Proteins** | **Fold-enrichment** | **FDR** |
| --- | --- | --- | --- | --- |
| Cellular component | Collagen trimer | 3 | 54.91 | 3.19E-03 |
|  | Collagen-containing ECM | 26 | 27.99 | 4.08E-26 |
|  | Extracellular matrix | 27 | 17.11 | 2.60E-22 |
|  | External encapsulating structure | 27 | 17.11 | 1.74E-22 |
|  | Extracellular space | 28 | 4.04 | 2.55E-08 |
|  | Extracellular region | 28 | 3.85 | 6.36E-08 |
|  | Cell periphery | 43 | 2.81 | 1.68E-08 |

**Table S8:** GO annotation of core enrichment proteins in hTCs +MMC. N = 3.

| **Category** | **Sub-category** | **Proteins** | **Fold-enrichment** | **FDR** |
| --- | --- | --- | --- | --- |
| Cellular component | Collagen-containing ECM | 28 | 26.73 | 1.53E-27 |
|  | Microtubule plus-end | 3 | 25.77 | 2.27E-02 |
|  | Extracellular matrix | 32 | 17.97 | 3.25E-27 |
|  | External encapsulating structure | 32 | 17.97 | 2.17E-27 |
|  | Extracellular space | 39 | 4.99 | 5.29E-15 |
|  | Extracellular region | 40 | 4.87 | 5.31E-15 |
|  | Supramolecular fiber | 9 | 4.04 | 2.93E-02 |
|  | Supramolecular polymer | 9 | 4.03 | 2.67E-02 |
|  | Supramolecular complex | 10 | 3.3 | 4.46E-02 |
|  | Cell periphery | 43 | 2.49 | 7.39E-07 |
|  | Cellular anatomical entity | 99 | 1.24 | 4.26E-02 |
|  | Cellular component | 101 | 1.24 | 3.94E-02 |

**Table S9:** GO annotation of core enrichment proteins in hDFs PLLA. N = 3.

| **Category** | **Sub-category** | **Proteins** | **Fold-enrichment** | **FDR** |
| --- | --- | --- | --- | --- |
| Cellular component | Microtubule plus-end | 3 | 21.89 | 1.50E-02 |
|  | Collagen-containing ECM | 15 | 12.16 | 3.04E-09 |
|  | Myofibril | 5 | 10 | 6.54E-03 |
|  | Contractile fiber | 5 | 9.69 | 7.04E-03 |
|  | Sarcomere | 4 | 9.19 | 3.27E-02 |
|  | Extracellular matrix | 17 | 8.11 | 2.28E-08 |
|  | External encapsulating structure | 17 | 8.11 | 1.52E-08 |
|  | Actin cytoskeleton | 10 | 5.56 | 1.33E-03 |
|  | Supramolecular fiber | 14 | 5.34 | 6.09E-05 |
|  | Supramolecular polymer | 14 | 5.33 | 5.26E-05 |
|  | Supramolecular complex | 18 | 5.04 | 4.10E-06 |
|  | Polymeric cytoskeletal fiber | 10 | 4.98 | 1.90E-03 |
|  | Cytoskeleton | 19 | 3.04 | 1.29E-03 |
|  | Extracellular space | 23 | 2.5 | 2.09E-03 |
|  | Extracellular region | 24 | 2.48 | 2.28E-03 |
|  | Intracellular non-membrane-bounded organelle | 28 | 2.27 | 2.16E-03 |
|  | Non-membrane-bounded organelle | 28 | 2.27 | 1.97E-03 |
|  | Cell periphery | 38 | 1.87 | 4.35E-03 |

**Table S10:** GO annotation of core enrichment proteins in hDFs +TGFB2. N = 3.

| **Category** | **Sub-category** | **Proteins** | **Fold-enrichment** | **FDR** |
| --- | --- | --- | --- | --- |
| Cellular component | Collagen trimer | 3 | 37.1 | 5.91E-03 |
|  | Collagen-containing ECM | 28 | 20.37 | 4.35E-24 |
|  | Stress fiber | 3 | 13.91 | 4.74E-02 |
|  | Actomyosin | 3 | 13.36 | 4.75E-02 |
|  | Extracellular matrix | 31 | 13.27 | 4.05E-22 |
|  | External encapsulating structure | 31 | 13.27 | 2.70E-22 |
|  | Myofibril | 5 | 8.98 | 1.24E-02 |
|  | Contractile fiber | 5 | 8.7 | 1.32E-02 |
|  | Sarcomere | 4 | 8.25 | 4.59E-02 |
|  | Actin cytoskeleton | 12 | 5.99 | 6.78E-05 |
|  | Supramolecular fiber | 15 | 5.14 | 2.58E-05 |
|  | Supramolecular polymer | 15 | 5.12 | 2.38E-05 |
|  | Supramolecular complex | 19 | 4.77 | 2.41E-06 |
|  | Extracellular region | 40 | 3.71 | 9.76E-11 |
|  | Extracellular space | 38 | 3.71 | 3.44E-10 |
|  | Cytoskeleton | 20 | 2.87 | 1.34E-03 |
|  | Cell junction | 14 | 2.53 | 4.57E-02 |
|  | Cell periphery | 56 | 2.48 | 8.64E-09 |
|  | Intracellular non-membrane-bounded organelle | 27 | 1.97 | 3.27E-02 |
|  | Non-membrane-bounded organelle | 27 | 1.97 | 3.06E-02 |

**Table S11:** GO annotation of core enrichment proteins in hDFs +MMC. N = 3.

| **Category** | **Sub-category** | **Proteins** | **Fold-enrichment** | **FDR** |
| --- | --- | --- | --- | --- |
| Cellular component | Microtubule plus-end | 3 | 24.23 | 1.90E-02 |
|  | Collagen-containing ECM | 17 | 15.25 | 3.13E-12 |
|  | Extracellular matrix | 20 | 10.56 | 3.80E-12 |
|  | External encapsulating structure | 20 | 10.56 | 2.53E-12 |
|  | Polymeric cytoskeletal fiber | 8 | 4.41 | 2.58E-02 |
|  | Supramolecular fiber | 10 | 4.22 | 1.03E-02 |
|  | Supramolecular polymer | 10 | 4.21 | 9.40E-03 |
|  | Supramolecular complex | 13 | 4.03 | 2.31E-03 |
|  | Extracellular space | 30 | 3.61 | 1.11E-07 |
|  | Extracellular region | 31 | 3.55 | 1.04E-07 |
|  | Cell periphery | 36 | 1.96 | 5.62E-03 |
